# Respiration Drives Dynamic Metal–Organic Framework for Smart Photoresponse to Volatile Toxic Vapors and Their Photodynamic Sterilization

**DOI:** 10.1002/advs.202501824

**Published:** 2025-05-20

**Authors:** Yun‐Lan Li, Hai‐Ling Wang, Ju‐Fen Ai, Guan‐Huang Zhang, Hua‐Hong Zou, Fu‐Pei Liang, Zhong‐Hong Zhu

**Affiliations:** ^1^ School of Chemistry and Chemical Engineering Guangxi Key Laboratory of Electrochemical Energy Materials Guangxi University Nanning 530004 P. R. China; ^2^ School of Chemistry and Pharmaceutical Sciences State Key Laboratory for Chemistry and Molecular Engineering of Medicinal Resources Guangxi Normal University Guilin 541004 P. R. China

**Keywords:** aggregation‐induced emission, dynamic metal–organic framework, photodynamic sterilization, smart photoresponse, solvothermal synthesis

## Abstract

Using aggregation‐induced emission luminous (AIEgens) containing dynamic molecular rotor structures as linkers to construct flexible smart luminescent metal–organic frameworks (MOFs) has become a transformative approach to constructing artificial intelligence color‐changing materials. Herein, 4′,4″,4′″,4″″‐(ethene‐1,1,2,2‐tetrayl)tetrabiphenyl‐4‐carboxylic acid (H_4_TPPE) is selected as a linker, and octahedral Zr_6_O_4_(OH)_8_(H_2_O)_4_ cluster are used as secondary building unit (SBU) to construct the first smart luminescent MOF (Zr‐TPE‐MOF) that can be driven by CH_2_Cl_2_ or CH_3_COOH vapor for respiration. It is worth noting that Zr‐TPE‐MOF can absorb trace amounts of CH_2_Cl_2_ or CH_3_COOH vapor into the pores through respiration and shows a blue shift of the emission wavelength up to 479 nm and an increase of emission intensity by nearly three times. In addition, the thermochromic behavior of Zr‐TPE‐MOF is not obvious in the temperature range of 80–350 K, but it has obvious thermofluorochromics behavior in the temperature range of 350–470 K. Zr‐TPE‐MOF showed highly sensitive and visualized smart photoresponse to the highly toxic Cr_2_O_7_^2−^, with a detection limit as low as 7.49 µm. Benefiting from the porous framework structure and organic–inorganic hybrid characteristics of Zr‐TPE‐MOF, it has excellent ROS generation ability and has excellent application prospects in photodynamic sterilization and rapid degradation of colored dyes.

## Graphical abstract

The flexible AIEgen (H_4_TPPE) with obvious molecular rotor structure is used as a linker to connect with the octahedral Zr_6_O_4_(OH)_8_(H_2_O)_4_ cluster‐based SBU to obtain the first smart luminescent MOF (Zr‐TPE‐MOF) with dynamic respiration driven by toxic vapors.

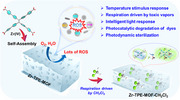

## 1 Introduction

With the rapid development of the economy, the problem of environmental pollution has become increasingly prominent, especially the toxic gases emitted by industrial production activities, such as acetic acid, dichloromethane, nitrogen oxides, difficult‐to‐degrade dark dyes, and heavy metal ions, which have continuously impacted the ecological balance through various environmental media and have threatened human health.^[^ ^1^, ^2^, ^3^, ^4^, ^5^ ^]^ Therefore, it is crucial to develop highly sensitive, easy‐to‐operate, and highly visualized sensors for monitoring environmental pollutants that are harmful to humans.^[^ ^6^, ^7^, ^8^ ^]^ Metal–organic frameworks (MOFs), porous crystalline materials formed by self‐assembly of metal ions or metal clusters and organic ligands through coordination bonds, have shown great advantages in catalysis, luminescence, gas storage, and diagnosis and treatment due to their high porosity, large specific surface area, rich active sites, and easy structural modification.^[^ ^9^, ^10^, ^11^, ^12^, ^13^, ^14^, ^15^, ^16^, ^17^, ^18^, ^19^, ^20^, ^21^ ^]^ As one of the important branches of metal–organic frameworks, luminescent metal–organic frameworks (LMOFs) not only inherit the general properties of MOFs but also exhibit highly visualized photoluminescence properties, which has promoted LMOFs to receive widespread attention in the fields of visualized and highly sensitive intelligent optical sensing and photocatalysis.^[^ ^22^, ^23^, ^24^, ^25^, ^26^, ^27^, ^28^, ^29^, ^30^ ^]^ The light‐emitting sources of LMOFs with organic–inorganic hybrid and porous properties can be organic linkers, guest molecules, or secondary building units (SBUs, usually metal ions or metal clusters).^[^ ^31^, ^32^ ^]^ The organic linkers for constructing LMOFs usually select traditional fluorophores as the parent core (for example, derivatives of traditional fluorophores such as quinoline, phenanthroline, pyrene, anthracene, and perylene diimide), and further modify suitable and symmetrical coordination sites or coordination pockets, thus becoming common and reliable organic linkers.^[^ ^33^, ^34^, ^35^ ^]^ These traditional fluorophore cores all have large planar structures, which results in the obtained LMOFs having a rigid “static” framework structure, and it is difficult to show rich and dynamic luminescence change behavior.^[^ ^36^, ^37^ ^]^

Molecular dynamics are ubiquitous in biology, with many biomolecular machines such as ATP synthase, ribosomes, and myosin converting energy into continuous and complex structural motions.^[^ ^38^, ^39^, ^40^, ^41^ ^]^ Similarly, introducing dynamic elements or dynamic modules as organic connecting pillars into LMOF structures to obtain artificial intelligence molecular machines is expected to become a transformative approach for designing new dynamic intelligent framework materials.^[^ ^42^, ^43^, ^44^, ^45^ ^]^ Over the past two decades, aggregation‐induced emission luminous (AIEgens) containing molecular rotors or vibrational units have provided opportunities for constructing dynamic smart optical materials with excellent photophysical properties.^[^ ^46^, ^47^, ^48^ ^]^ When AIEgens are used as organic linkers to construct LMOFs, the restriction of intramolecular motions (RIMs) of the molecular rotors within the framework can help LMOFs display high fluorescence brightness.^[^ ^46^, ^47^, ^48^ ^]^ Micro‐perturbation of AIEgens by specific guest molecules can exhibit fast optical response behavior, thus achieving highly sensitive chemical sensing.^[^ ^45^, ^49^, ^50^, ^51^ ^]^ In addition, twisted AIEgens contribute to the formation of novel topological structures of LMOFs, and the precise structural information of LMOFs also provides a guarantee for the study of the RIM mechanism of AIEgens.^[^ ^52^, ^53^ ^]^ In recent years, AIEgens based on tetraphenylethylene and triphenylamine as the core have been used to synthesize AIEgens‐based LMOFs with organic linkers.^[^ ^54^, ^55^ ^]^ In 2021, Tang et al. used TPE tetracarboxylic acid derivatives (TCPE) to construct two AIE‐MOFs (ZnMOF and CoMOF) with uneven charge distribution and successfully achieved a sensitive response of fluorescence and molecular magnetism to HCl gas.^[^ ^56^ ^]^ In 2024, Chen et al. designed and synthesized four new zirconium‐based metal–organic frameworks (Zr‐MOFs) through a ligand desymmetrization strategy.^[^ ^57^ ^]^ These Zr‐MOFs exhibited unique structural diversity and efficient adsorption performance for toxic chemicals. Although abundant AIEgens‐based LMOFs have been obtained by alternately connecting diverse AIEgens as organic linkers with SBUs, research on the flexibility of dynamic motifs within the AIEgens‐based LMOFs structure is still very scarce.^[^ ^54^ ^]^ The breathing of AIEgens‐based LMOFs is an important feature that demonstrates the flexibility of the framework. Using guest molecules in the pores to drive the breathing of AIEgens‐based LMOFs opens new horizons for the development of flexible materials for artificial intelligence.^[^ ^54^, ^55^, ^58^ ^]^ It is even rarer to be able to drive the respiration of AIEgens‐based LMOFs by highly toxic vapors, thereby displaying highly sensitive smart photoresponsive behaviors.^[^ ^56^ ^]^

Herein, the flexible tetradentate carboxylate ligand H_4_TPPE was used to construct the first AIEgens‐based LMOFs (Zr‐TPE‐MOF) with dynamic respiration driven by toxic vapors. It is worth noting that after the dynamic molecular rotor was introduced into the framework structure, Zr‐TPE‐MOF exhibited sensitive thermofluorochromics behavior only in the high‐temperature environment. Its fluorescence intensity decreased with the increase in temperature, and the wavelength red‐shifted. In addition, Zr‐TPE‐MOF can rapidly absorb trace amounts of CH_2_Cl_2_ or CH_3_COOH vapor and exhibit highly sensitive intelligent photoresponsive behavior, thus developing an intelligent photoresponsive sensor for volatile toxic organic compounds. In addition, Zr‐TPE‐MOF also exhibited highly sensitive smart photoresponse to Cr_2_O_7_^2−^ ions with a limit of detection (LOD) as low as 7.49 µm, which is significantly better than most optical sensing materials. At the same time, the portable test strips obtained after simple preparation of Zr‐TPE‐MOF can realize visual real‐time detection of extremely low concentrations of Cr_2_O_7_^2−^ ions in aqueous solution. Moreover, Zr‐TPE‐MOF with excellent ROS production ability under light irradiation conditions can effectively inhibit the rapid proliferation of *Escherichia coli* (*E. coli*) and *Staphylococcus aureus* (*S. aureus*) and photocatalytically degrade dark organic dyes. To the best of our knowledge, this is the first time that AIEgens‐based LMOFs have been shown to exhibit intelligent photoresponsive behavior by dynamic respiration driven by toxic vapors. This work not only opens a door for designing LMOFs with dynamic respiration but also opens new horizons for the rational construction of artificial intelligence porous materials.

## 2 Results and Discussion

### 2.1 Structural Analysis of Zr‐TPE‐MOF

**Figure 1:**
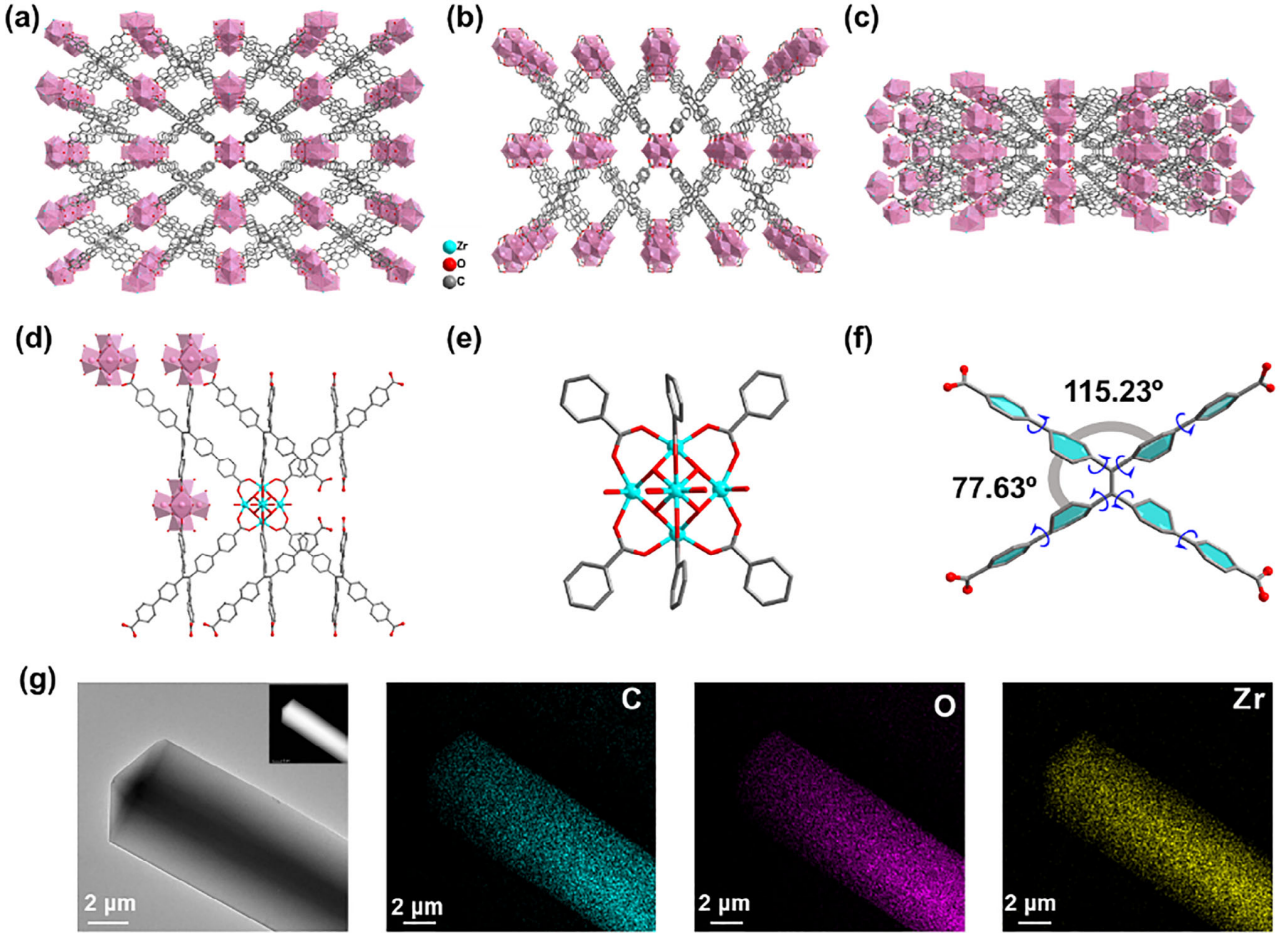
a–c) The structures of Zr‐TPE‐MOF in different directions; d) The connection diagram of AIEgen TPPE^4^^−^ as a linker with four Zr_6_ cluster‐based SBUs; e) The structure of Zr_6_ cluster‐based SBU; f) The twisting angle between the benzene rings of AIEgen TPPE^4^^−^ in the Zr‐TPE‐MOF structure; g) HR‐TEM image and EDS element mapping results of Zr‐TPE‐MOF.

The AIEgen 4′,4″,4′″,4″″‐(ethene‐1,1,2,2‐tetrayl)tetrabiphenyl‐4‐carboxylic acid (H_4_TPPE) with a molecular rotor structure reacted with ZrOCl_2_ under solvothermal conditions to obtain colorless and transparent crystalline Zr‐TPE‐MOF. Single crystal X‐ray diffraction (SCXRD) results show that Zr‐TPE‐MOF crystallizes in the *Fmmm* space group of the orthorhombic system (Table S1, Supporting information). The molecular rotor ligand TPPE^4^^−^ in the Zr‐TPE‐MOF structure connects four octahedral secondary building units (SBUs) Zr_6_O_4_(OH)_8_(H_2_O)_4_ clusters that are in different planes and have the same structural connections (**Figure** **1**). Each SBU is connected to the ‐COO^−^ in the molecular rotor TPPE^4^^−^ structure at the vertex position. The pores in the Zr‐TPE‐MOF structure have two rhombus‐shaped 1D pores, and the TPE derivative TPPE^4^^−^ with an elongated molecular rotor structure gives Zr‐TPE‐MOF a larger pore cavity (Figure 1a–c). The benzene rings of the molecular rotor TPPE^4^^−^ in the Zr‐TPE‐MOF structure can rotate freely in sufficient pore cavities, and the dihedral angles between the benzene rings on the same side are 77.63°, and the dihedral angles between the benzene rings on different sides are 115.23° (Figure 1f). The TPPE^4^^−^ ligand of the extended molecular rotor has a certain degree of flexibility, which can endow Zr‐TPE‐MOF with host–guest interactions that are highly sensitive to changes in guest molecules within the pores. High‐resolution transmission electron microscopy (HR‐TEM) results show that Zr‐TPE‐MOF is a bulk crystal with a very clean surface (Figure 1g; Figure S1, Supporting information). In addition, elemental mapping results show that the C, O, and Zr elements in the Zr‐TPE‐MOF structure are very uniformly distributed.

The Fourier transform infrared (FTIR) absorption spectrum of Zr‐TPE‐MOF showed obvious absorption peaks at 3400, 1600, and 1400 cm^−1^, respectively (Figure S2, Supporting information). The absorption peak at ≈3400 cm^−1^ corresponds to the vibration of *ν*(HO─H) in the H_2_O molecule or ─OH group, the absorption peak at ≈1600 cm^−1^ is attributed to the vibration of *ν*(C═O) in the carboxylic acid group, and the absorption peak at ≈1400 cm^−1^ can be attributed to the stretching vibration of *ν*(C−C) in the benzene ring.^[^ ^59^ ^]^ Thermogravimetric (TG) tests of Zr‐TPE‐MOF were performed in a nitrogen atmosphere at a heating rate of 5 °C min^−1^ from 35 to 1000 °C (Figure S3, Supporting information). The Zr‐TPE‐MOF lost 5.8% of its weight before 90 °C, corresponding to the loss of one *N*,*N*‐diethylformamide (DEF) in the pores (theoretical weight: 5.5%). Furthermore, the temperature continued to increase to 274 °C, and the weight loss of Zr‐TPE‐MOF was 28.6%, corresponding to the loss of five DEF molecules in the pores (theoretical weight: 28.9%). The observed and simulated values of powder X‐ray diffraction (PXRD) of Zr‐TPE‐MOF are highly consistent, proving that the large number of Zr‐TPE‐MOF crystals obtained are pure phase (Figure S4, Supporting information). For a long time, stability has been considered a key issue in the practical application of MOFs.^[^ ^60^, ^61^ ^]^ For smart MOFs with molecular rotors, stability research is particularly important. Therefore, to explore the stability of Zr‐TPE‐MOF, it was immersed in various solvents (DMSO, DMF, MeOH, MeCN, and EtOH) and aqueous solutions with different pH values (pH 3, 5, 7, 9, and 11) for 12 h and then taken out for PXRD testing. It is worth noting that Zr‐TPE‐MOF still maintains high stability after being immersed in different solvents and pH aqueous solutions for 12 h (Figure S5, Supporting information). Therefore, Zr‐TPE‐MOF can be considered a very rare example of a highly stable smart dynamic MOF.

### 2.2 Photophysical Properties of Zr‐TPE‐MOF

**Figure 2:**
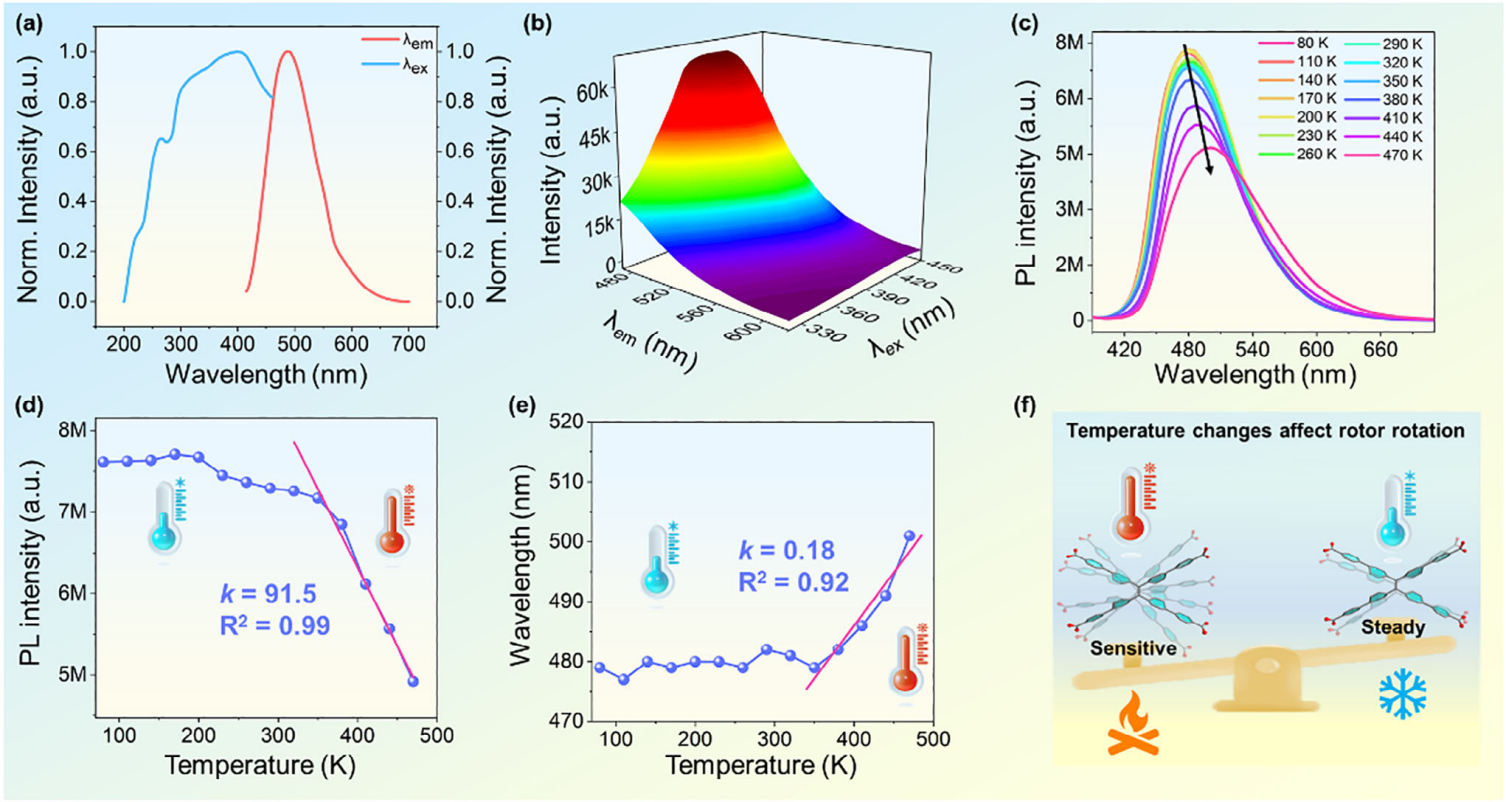
a) Excitation and emission spectra of Zr‐TPE‐MOF; b) 3D fluorescence spectrum of Zr‐TPE‐MOF; c–e) Variable temperature fluorescence spectrum of Zr‐TPE‐MOF, emission peak intensity, and wavelength curves as the temperature changes; f) Schematic diagram of the temperature‐induced change in the degree of motion of the molecular rotors in the Zr‐TPE‐MOF structure, which leads to luminescence changes.

Under 365 nm UV light, bright yellow‐green luminescence was observed for the dried Zr‐TPE‐MOF. Therefore, the photophysical properties of Zr‐TPE‐MOF were further explored. Specifically, the fluorescence emission curve of solid‐state Zr‐TPE‐MOF was tested using 390 nm excitation, which showed a broad emission peak with a maximum wavelength at 485 nm (**Figure** **2**a). Similarly, the 3D fluorescence spectrum also demonstrated that the optimal excitation and emission peaks of Zr‐TPE‐MOF are located at 390 and 480 nm, respectively (Figure 2b). At the same time, the liquid 3D fluorescence spectrum of Zr‐TPE‐MOF dispersed in a mixed solution of DMSO: H_2_O (1: 99) also showed that the optimal excitation and emission peaks of Zr‐TPE‐MOF were consistent with the solid‐state 3D fluorescence spectrum (Figure S6, Supporting information). In addition, the solid‐state fluorescence quantum yield of Zr‐TPE‐MOF is as high as 50.5%, and the fluorescence lifetime is 2.58 ns (Figure S7a,d, Supporting information). The above series of photophysical properties prove that Zr‐TPE‐MOF has reliable fluorescence emission behavior, which can further explore its application prospects in the field of optics. Next, to further explore and analyze the movement of the molecular rotor ligand TPPE^4^^−^ within the structure of Zr‐TPE‐MOF, the variable temperature fluorescence spectra of Zr‐TPE‐MOF were tested in the temperature range of 80—470 K (Figure 2c). As the temperature gradually increases, the emission peak of Zr‐TPE‐MOF red‐shifts, and the intensity decreases, proving that the degree of motion of the molecular rotor intensifies with increasing temperature (Figure 2d). The fluorescence intensity and wavelength of Zr‐TPE‐MOF were further plotted as a function of temperature (Figure 2e). It is clearly shown that in the low‐temperature range (80—350 K), the fluorescence emission intensity and wavelength of Zr‐TPE‐MOF do not change significantly during the temperature rise process, indicating that the molecular rotor motion of Zr‐TPE‐MOF is not sensitive in the temperature range of 80—350 K. It is worth noting that the fluorescence emission intensity and wavelength of Zr‐TPE‐MOF change significantly in the temperature range of 350—470 K, and its emission peak intensity decreases from ≈6.9 × 10^6^ (a.u.) to ≈4.1 × 10^6^ (a.u.), and the emission wavelength red‐shifted from 479 to 500 nm (Figure 2e). The above results show that the movement of the molecular rotor ligand TPPE^4^^−^ in the Zr‐TPE‐MOF pore is affected by temperature changes, and the movement of TPPE^4^^−^ is more obvious in the high‐temperature zone, which can lead to obvious changes in fluorescence emission (Figure 2f). To further verify the stability of Zr‐TPE‐MOF in the high‐temperature region, its PXRD was tested in the temperature range of 320–440 K. With the increase of temperature, the PXRD observed values of Zr‐TPE‐MOF are highly consistent with their theoretical values, proving that Zr‐TPE‐MOF has high stability in the above temperature range (Figure S8, Supporting information). Generally, dynamic MOFs containing molecular rotors are expected to show temperature‐dependent changes in emission spectra, but dynamic MOFs with highly sensitive photoresponse only in the high‐temperature region are still very rare.

### 2.3 Specific Host–Guest Interaction Induces the Respiration of Zr‐TPE‐MOF

**Figure 3:**
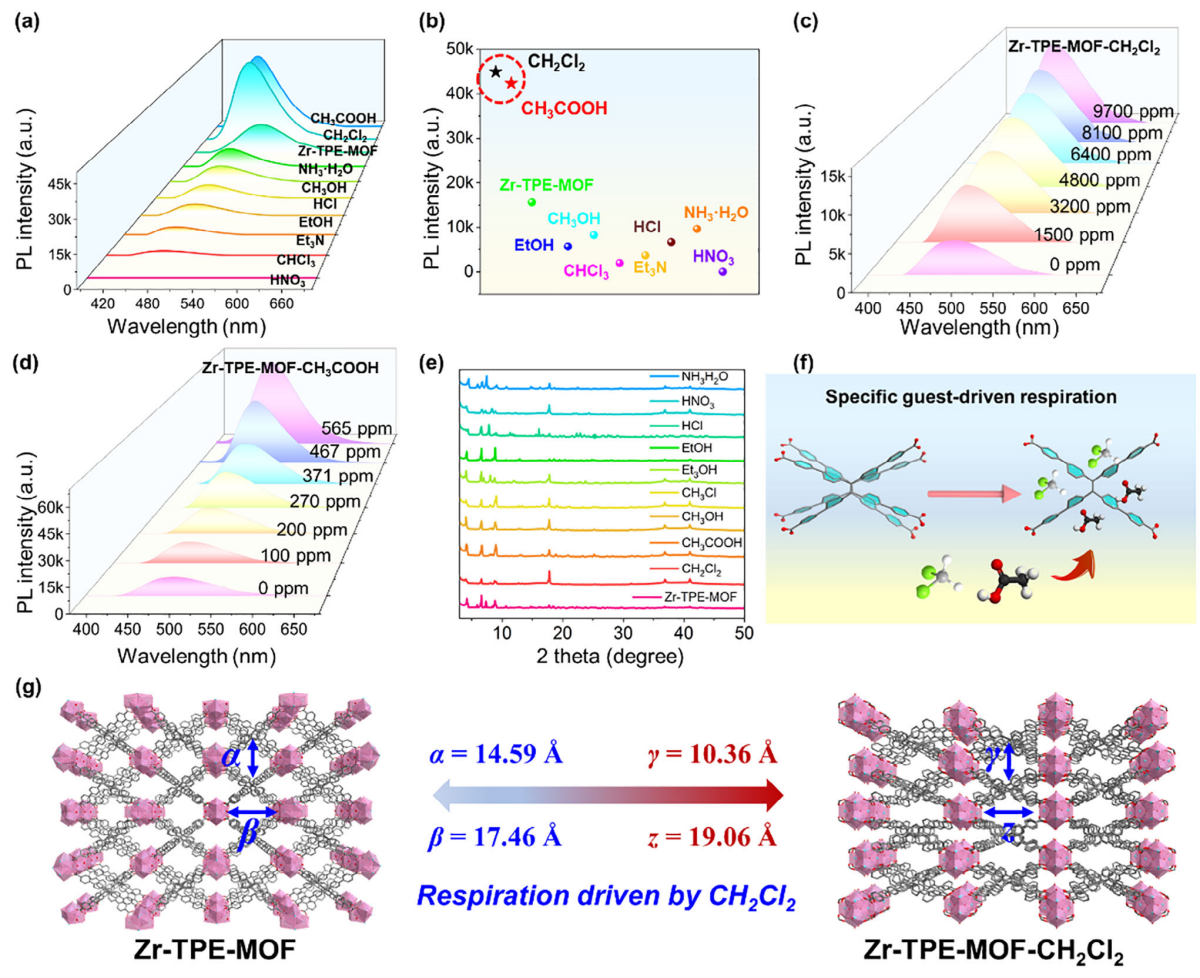
a,b) Comparison of emission spectra and intensity after fumigation of Zr‐TPE‐MOF with different volatile solvents; c,d) Fluorescence spectra changes caused by fumigation of Zr‐TPE‐MOF with different concentrations of CH_2_Cl_2_ or CH_3_COOH vapor; e) PXRD of Zr‐TPE‐MOF after fumigation of Zr‐TPE‐MOF with different volatile solvents; f) Schematic diagram of CH_2_Cl_2_ or CH_3_COOH vapor entering the pores of Zr‐TPE‐MOF to induce respiration through host–guest interaction; g) Changes in pore size before and after CH_2_Cl_2_ vapor enters the pores of Zr‐TPE‐MOF.

Based on the fact that changes in the degree of movement of the molecular rotor within the porous material framework can lead to significant changes in fluorescence emission, volatile toxic gases were further utilized to deeply explore the smart photoresponsive properties of Zr‐TPE‐MOF. Toxic vapors emitted by HNO_3_, CHCl_3_, Et_3_N, EtOH, HCl, MeOH, NH_3_·H_2_O, CH_2_Cl_2,_ and CH_3_COOH were selected to fumigate Zr‐TPE‐MOF, and the emission spectrum was used to track the changes in the emission spectrum of Zr‐TPE‐MOF after stimulation by the above‐mentioned stimulus factors (**Figure** **3**a). Obvious fluorescence enhancement and blue shift behavior were observed in Zr‐TPE‐MOF after being smoked with CH_2_Cl_2_ and CH_3_COOH respectively (Figure 3a). The fluorescence emission intensity of Zr‐TPE‐MOF increased by 2.87 and 2.71 times after being smoked with CH_2_Cl_2_ and CH_3_COOH, respectively (Figure 3b). The lifetimes of Zr‐TPE‐MOF after absorbing CH_2_Cl_2_ and CH_3_COOH are 1.59 and 1.61 ns, respectively, and the fluorescence quantum yields (QY) are 60.5% and 61.5%, respectively (Figure S7b,c,e,f, Supporting information). Compared with Zr‐TPE‐MOF, the QY after imbibing CH_2_Cl_2_ vapor was enhanced by 1.52 times. In addition, Zr‐TPE‐MOF was fumigated with CH_2_Cl_2_ and CH_3_COOH with different vapor concentrations, and the changes in the fluorescence emission of Zr‐TPE‐MOF after fumigation were tested. Finally, the limits of detection (LOD) of Zr‐TPE‐MOF for CH_2_Cl_2_ and CH_3_COOH were obtained to be 755.45 and 7.13 ppm, respectively (Figure 3c,d; Figure S9, Supporting information). It is worth noting that the porous framework structure of Zr‐TPE‐MOF still maintains high stability after being smoked by the above different factors (Figure 3e). In addition, Zr‐TPE‐MOF after CH_2_Cl_2_ and CH_3_OOH vapor fumigation was selected for UV–vis diffuse reflectance spectroscopy testing. The results showed that the absorption peak position of Zr‐TPE‐MOF after different vapor fumigation was the same as that of untreated Zr‐TPE‐MOF, which further proved that Zr‐TPE‐MOF has excellent stability (Figure S10, Supporting information). The Zr‐TPE‐MOF after CH_2_Cl_2_ vapor fumigation was selected for SCXRD testing, and the results showed that a small amount of CH_2_Cl_2_ entered its pores (Figure 3f). The CH_2_Cl_2_ entering Zr‐TPE‐MOF not only disturbed the motion of the molecular rotor but also induced changes in the unit cell. More importantly, the absorption of CH_2_Cl_2_ into Zr‐TPE‐MOF induced a significant change in the size of the pores, with the length and width of the pores changing from 14.59 and 17.45 Å to 10.36 and 19.06 Å (Figure 3g; Figure S11, Supporting information). The above results clearly show that the CH_2_Cl_2_ vapor guest in the pores can induce Zr‐TPE‐MOF to have obvious breathing, thereby enhancing its intelligent photoresponsive behavior to CH_2_Cl_2_. In recent years, a lot of research has been devoted to the development of luminescent metal–organic framework materials (LMOFs) for detecting toxic gases emitted by industrial production. This type of material uses different fluorophores as organic linkers to alternately connect to secondary building units (SBUs), and uses the interaction between fluorophores and gas molecules to detect and identify target gases through changes in fluorescence signals. Although LMOFs have shown significant advantages in the field of toxic gas detection, they still face challenges in practical applications such as insufficient selectivity, limited sensitivity, poor stability, and long response time. This is mainly because the optical properties of LMOFs rely on traditional static planar rigid ligands. These rigid ligands are poorly sensitive to external stimuli and have weak host–guest interactions, making it difficult for them to exhibit highly sensitive sensing behavior. To address the above issues, we used a derivative of tetraphenylethylene core (H_4_TPPE) with an extended molecular rotor structure as a linker to obtain Zr‐TPE‐MOF, which can exhibit highly sensitive intelligent photoresponse behavior to CH_2_Cl_2_ and CH_3_COOH vapors through the “breathing” effect. Compared with traditional LMOFs, toxic gases entering the pores of the dynamic Zr‐TPE‐MOF can effectively interfere with the movement of the molecular rotors and cause significant changes in the degree of distortion of the molecular rotors, thereby inducing rapid changes in their optical properties. As far as we know, this is the first time that the dynamic breathing of MOFs containing molecular rotor structures induced by volatile toxic vapors has been discovered, opening up a new blueprint for the construction of stimulus‐responsive artificial intelligence porous materials.

### 2.4 Zr‐TPE‐MOF Intelligent Light Response to Heavy Metal Ions Cr_2_O_7_^2−^

**Figure 4:**
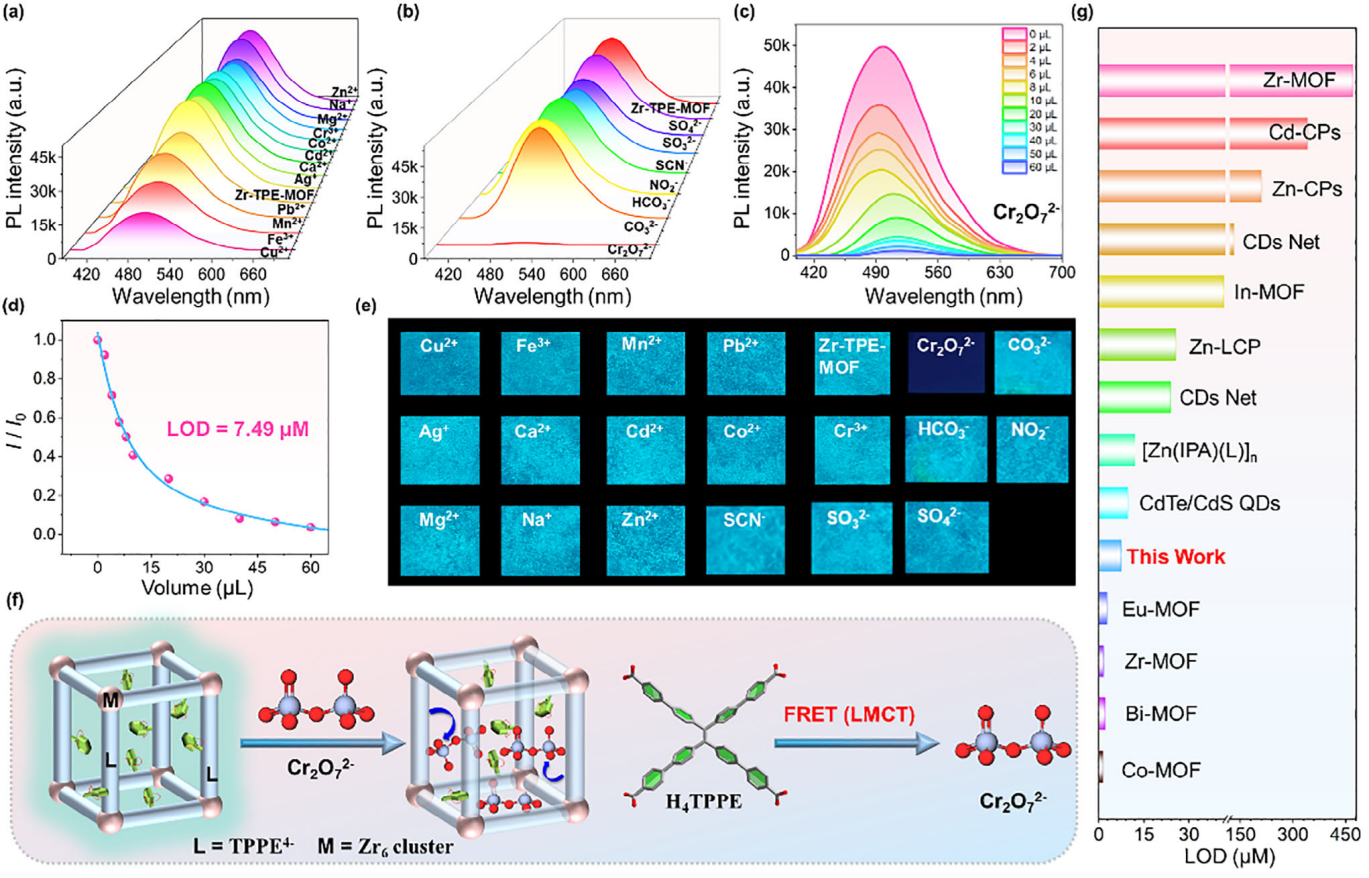
a,b) Emission spectra of Zr‐TPE‐MOF aqueous solutions containing different types of cations and anions; c,d) Changes in the fluorescence emission peak and detection limit curve after adding different concentrations of Cr_2_O_7_^2−^ into Zr‐TPE‐MOF aqueous solutions; e) The test paper strips after being immersed in Zr‐TPE‐MOF aqueous solutions respond intelligently to different types of cations and anions; f) Schematic diagram of the fluorescence quenching caused by Cr_2_O_7_^2−^ entering Zr‐TPE‐MOF, TPPE^4−^ and Cr_2_O_7_^2−^ undergo LMCT, which leads to fluorescence quenching; g) Comparison of the sensing performance of different types of sensing materials and Zr‐TPE‐MOF on Cr_2_O_7_^2−^.

Heavy metal ions are serious toxic pollutants in water. Their negative effects on organisms and threats to human health make it very important to develop sensors that can efficiently and conveniently identify toxic heavy metals.^[^ ^1^, ^2^, ^3^, ^62^ ^]^ Based on the excellent photophysical properties of Zr‐TPE‐MOF, the intelligent optical sensing experiments of different types of cations (such as Zn^2+^, Mg^2+^, Na^+^, Ca^2+^, Cr^2+^, Co^2+^, Cd^2+^, Ag^+^, Pd^2+^, Mn^2+^, Fe^3+^, Cu^2+^) and anions (SCN^−^, HCO_3_^−^, CO_3_^2−^, NO_2_^−^, SO_4_^2−^, SO_3_^2−^, Cr_2_O_7_^2−^) were explored. Aqueous solutions containing different types of cations and anions were added to the aqueous solution containing Zr‐TPE‐MOF, and the changes in the luminescence behavior of the mixed solution were observed under 365 nm ultraviolet light. The results show that the blue‐green fluorescence of Zr‐TPE‐MOF is quickly extinguished in the mixed solution containing Cr_2_O_7_^2−^, while the other experimental groups have obvious blue‐green fluorescence emissions. The emission spectrum of the above solutions was further tested, and the results showed that the emission of Zr‐TPE‐MOF in the experimental group containing the mixed solution of Cr_2_O_7_^2−^ ions disappeared significantly, while the other experimental groups still showed obvious Zr‐TPE‐MOF emission (**Figure** **4**a,b). To further explore the sensing sensitivity of Zr‐TPE‐MOF to Cr_2_O_7_^2−^ ions, photoluminescence titration experiments were carried out. Different volumes of aqueous solution of Cr_2_O_7_^2−^ ions with a concentration of 5 mg mL^−1^ (0, 2, 4, 6, 8, 10, 20, 30, 40, 50, 60 µL) were accurately measured and added to the Zr‐TPE‐MOF solution, and the emission spectra of the above solutions were tested in turn. The results showed that as the Cr_2_O_7_^2−^ ions concentration gradually increased, the blue luminescence displayed by Zr‐TPE‐MOF decreased significantly and eventually completely extinguished (Figure 4c). The detection limit of the above light response process was calculated according to the Stern‐Volmer equation *I_0_/I* = 1+*K*_sv_[M]. The calculation results show that the LOD of Zr‐TPE‐MOF for Cr_2_O_7_^2−^ is 7.49 µm (Figure 4d). In addition, the UV–vis spectra of Zr‐TPE‐MOF aqueous solution containing Cr_2_O_7_^2−^ and Zr‐TPE‐MOF aqueous solution were further tested, which clearly showed that the entry of Cr_2_O_7_^2−^ into Zr‐TPE‐MOF caused a significant shift in its absorption peak, from a broad absorption peak at 320 nm to a triple absorption peak at 293, 350, and 446 nm, proving that there is an obvious energy transfer between Cr_2_O_7_^2−^ and TPPE^4^^−^ (Figure S12, Supporting information). These results indicate that Zr‐TPE‐MOF has great potential as an efficient photoresponsive sensor for Cr_2_O_7_^2−^. We believe that the fluorescence quenching of Zr‐TPE‐MOF can be attributed to the interaction between the organic ligands of Zr‐TPE‐MOF and Cr_2_O_7_^2−^, accompanied by ligand‐to‐metal charge transfer (LMCT), which leads to fluorescence quenching. In addition, compared with most common luminescent materials (e.g., metal–organic framework Co/Zr/Cd/Eu/Bi‐MOF, quantum dots, nanorods, etc.), Zr‐TPE‐MOF exhibits more sensitive smart sensing performance to low‐concentration Cr_2_O_7_^2−^ ions (Figure 4g).^[^ ^63^, ^64^, ^65^, ^66^ ^]^ In summary, the above experimental results all show that Zr‐TPE‐MOF can efficiently, quickly, and conveniently monitor Cr_2_O_7_^2−^ ions in water, which is of great significance for detecting excessive toxic metal ions in water environments. In addition, portable test strips are popular due to their low cost, recyclability, convenience, high visualization, fast response, and environmental protection.^[^ ^62^, ^67^ ^]^ Based on the efficient and rapid intelligent response behavior of Zr‐TPE‐MOF to aqueous solutions of Cr_2_O_7_^2−^ ions, Zr‐TPE‐MOF was further expanded to be made into portable test strips. Specifically, we dispersed Zr‐TPE‐MOF in an aqueous solution with polyethylene glycol added and ultrasonically treated it to make a simple ink, which was then evenly coated on a cut block of ordinary filter paper to make a blank test paper. We dropped different types of metal ions and anions with the same concentrations onto blank test papers. Then, under the condition of 365 nm ultraviolet light, we could observe with the naked eye that the blue light of the original test paper containing the Cr_2_O_7_^2−^ ion solution disappeared, while the other test papers all emitted blue light, which was consistent with the color of the blank test paper without any solution added (Figure 4e).

### 2.5 Photodynamic Sterilization Activity of Zr‐TPE‐MOF

**Figure 5:**
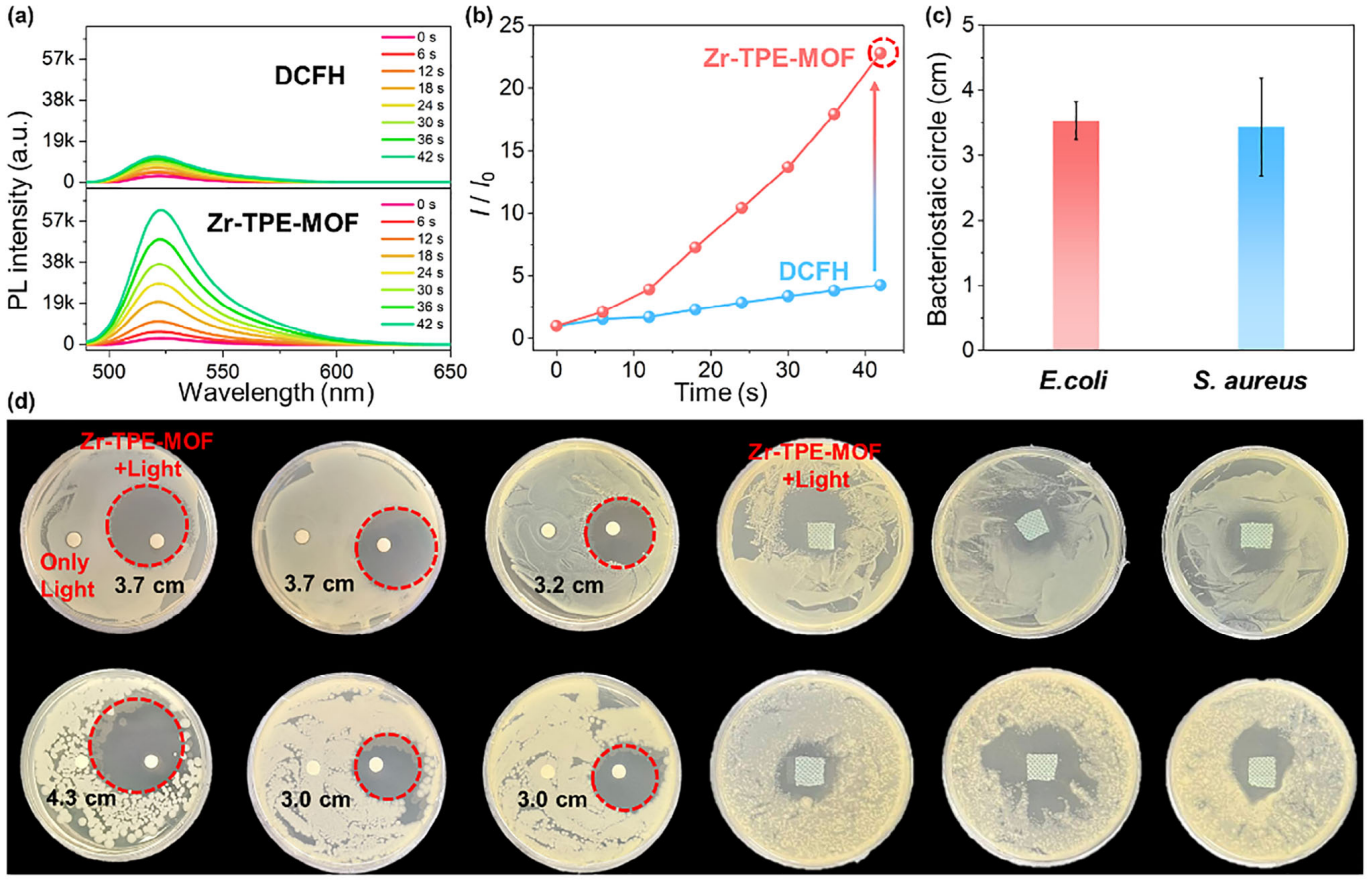
a) Fluorescence spectrum of DCFH (50 µm) in H_2_O/DMSO mixed solvent (*V*_water_/*V*_DMSO_ = 99:1) changes with illumination time; b) Fluorescence spectrum of Zr‐TPE‐MOF with a concentration of 10 µg mL^−1^ in H_2_O/DMSO mixed solvent (*V*_water_/*V*_DMSO_ = 99:1) containing DCFH (50 µm) changes with illumination time; c) The size of the inhibition zone of Zr‐TPE‐MOF against *E. coli* and *S. aureus* under illumination; d) Photodynamic sterilization effect of medical masks loaded with Zr‐TPE‐MOF on *E. coli* and *S. aureus* under illumination (60 mW cm^−2^, 5 min) (Repeat 3 times, *n*  =  3).

Based on the above photophysical properties, activated DCFH (DCFH‐DA activated by NaOH) was selected as an indicator of total ROS to monitor the ROS production capacity of Zr‐TPE‐MOF.^[^ ^68^, ^69^ ^]^ Under low power density (60 mW cm^−2^) light irradiation conditions, Zr‐TPE‐MOF can effectively enhance the fluorescence of the DCFH indicator, with a fluorescence enhancement factor of up to 23 times (**Figure** **5**a,b). The fluorescence enhancement factor of the DCFH control group under light irradiation at the same power density was negligible, demonstrating the excellent ROS generation ability of Zr‐TPE‐MOF (Figure 5a). To further explore the types of ROS produced by Zr‐TPE‐MOF under light irradiation, DHR 123 and HPF were selected as specific indicators to monitor the generation capabilities of ·O_2_^−^ and ·OH, respectively. Obviously, with the increase of 60 mW cm^−2^ light irradiation time, the characteristic emission of DHR 123 and HPF gradually enhanced, and the fluorescence enhancement factors were as high as 86 and 19 times, respectively, proving that Zr‐TPE‐MOF has an excellent ability to generate type I ROS (Figure S13, Supporting information). In addition, 9,10‐anthracenediyl‐bis(methylene)bismalonic acid (ABDA) was used to monitor the generation ability of type II ROS in Zr‐TPE‐MOF under light irradiation and dark conditions. The experimental results show that with the increase of 60 mW cm^−2^ light irradiation time, the characteristic absorption peak intensity of ABDA is significantly weakened, proving that Zr‐TPE‐MOF can efficiently generate type II ROS under light irradiation conditions (Figure S14, Supporting information). Obviously, the introduction of Zr_6_ cluster‐based SBU within the structure leads to a more obvious charge separation effect, promoting the excellent type I ROS generation ability of Zr‐TPE‐MOF. In addition, the large specific surface area and obvious porous structure lead to a significant increase in the contact area between Zr‐TPE‐MOF and oxygen molecules, thereby enhancing its ability to generate type II ROS. Based on the in‐depth exploration of photophysical properties and light‐driven ROS production capabilities, the application of Zr‐TPE‐MOF in the field of photodynamic sterilization has been further expanded. *E. coli* and *S. aureus* were selected to explore the photodynamic sterilization effect of Zr‐TPE‐MOF. The effects of different light irradiation times and powers on the performance of photodynamic sterilization were explored, and comparative experiments with different xenon lamp powers and illumination times were further designed. The results showed that with the increase of light irradiation time and power, the inhibition zone produced by photodynamic sterilization increased significantly and then remained unchanged (Figure S15, Supporting information). Based on the above results, a power of 60 mW cm^−2^ and an irradiation time of 5 min were selected as the most suitable conditions for photodynamic sterilization. Specifically, the strains were recovered after 24 h using a 37 °C biochemical incubator, then inoculated into LB liquid culture medium for culture, and finally diluted with 0.9% (w/v) saline to prepare suspensions of the two bacteria as 0.5 McMahon turbidity standards (≈2 × 10^8^ CFU mL^−1^) for use. A solution of Zr‐TPE‐MOF with a concentration of 3 mg mL^−1^ was prepared for use, and the drug‐sensitive paper sheets were immersed in the above solution for 30 s, then quickly taken out and dried. The light irradiation experimental group was irradiated under a xenon lamp at 60 mW cm^−2^ for 5 min and then transferred to an incubator for 24 h. The experimental results show that the drug‐sensitive paper containing Zr‐TPE‐MOF has obvious photodynamic sterilization performance against *E. coli* and *S. aureus* under light irradiation conditions (Figure 5d). The control group of drug‐sensitive paper strips treated with light irradiation alone was covered with bacteria (Figure S16, Supporting information), proving that light irradiation alone cannot produce ROS to kill bacteria. It is worth noting that despite the very short light irradiation time, Zr‐TPE‐MOF also exhibited a significant photodynamic sterilization effect. The average inhibition zone of Zr‐TPE‐MOF against *E. coli* and *S. aureus* was ≈3.53 and 3.43 cm (Figure 5c), respectively. In addition, the sterilization performance of Zr‐TPE‐MOF loaded on masks was further explored. The culture and concentration of the bacteria were the same as those in the above experiment. Accurately pipette 100 µL of *E. coli* and *S. aureus* and evenly spread them on the LA medium. Medical masks cut into 1 × 1 cm pieces were immersed in a Zr‐TPE‐MOF solution containing 3 mg mL^−1^. After 30 s, they were taken out and added to the solid culture medium coated with the bacterial solution. Then, the illumination experimental group was placed under a 60 mW cm^−2^ xenon lamp for 5 min and then placed in a biochemical incubator for 24 h (Figure 5d). The experimental results show that no infection of *E. coli* and *S. aureus* was observed in the masks soaked in Zr‐TPE‐MOF after light irradiation, and there were obvious antibacterial zones around the masks. The masks of the control group that were only treated with light irradiation were covered with bacteria, proving that masks treated with light irradiation alone did not have a sterilization effect (Figure S16, Supporting information). Zr‐TPE‐MOF with a porous framework structure composed of cluster‐based SBU is expected to become a new type of photodynamic sterilization material.

### 2.6 Photocatalytic Degradation of Organic Dyes by Zr‐TPE‐MOF

**Figure 6:**
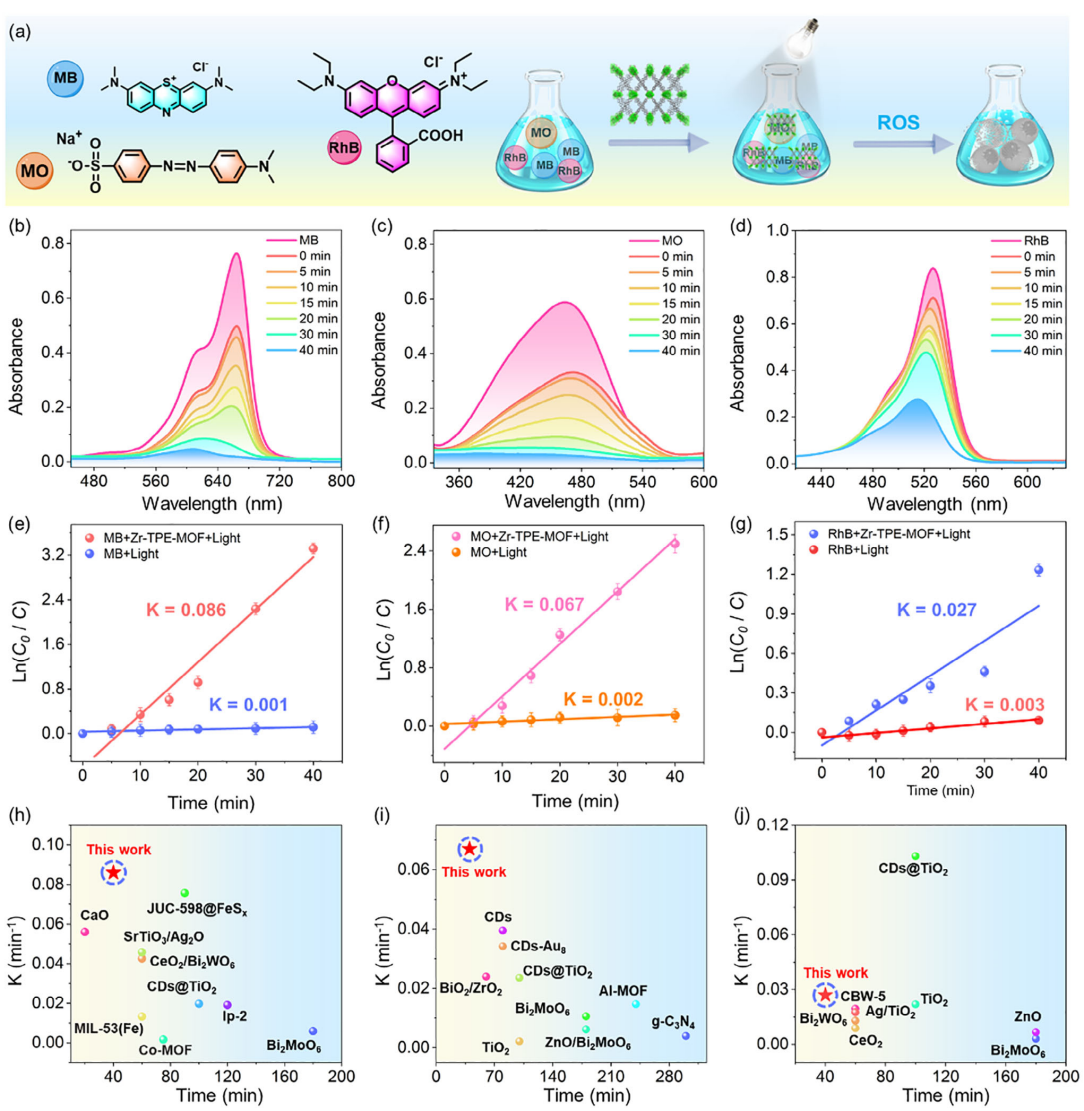
a) Schematic diagram of Zr‐TPE‐MOF generating ROS for degradation of organic dyes under light irradiation conditions; b–d) UV–vis absorption spectra of aqueous solutions (pH 7.0) containing MB, MO, and RhB (10 mg L^−1^) after adding 0.3 mg mL^−1^ Zr‐TPE‐MOF and treating with different light irradiation times (60 mW cm^−2^); e–g) Degradation kinetic fitting curves and degradation rates of 0.3 mg mL^−1^ Zr‐TPE‐MOF for MB, MO, and RhB, respectively (Repeat 3 times, *n*  =  3); h–j) Performance comparison of 0.3 mg mL^−1^ Zr‐TPE‐MOF and different types of photocatalytic materials reported so far in the field of degradation of organic dyes.

Water pollution has become a serious global problem, especially dark organic dye pollutants, which pose a major threat to the environment and human health. Traditional methods for treating organic dye pollutants face many limitations, such as high cost, low ecological efficiency, and easy to cause secondary pollution.^[^ ^1^, ^2^, ^3^, ^4^, ^5^ ^]^ In recent years, the photocatalytic system has been continuously expanded, and photosensitizers such as graphene, metal–organic framework materials (MOFs), and porous organic polymers (POPs) have shown great potential in the field of photocatalytic water purification.^[^ ^68^, ^69^, ^70^ ^]^ Based on the fact that Zr‐TPE‐MOF can generate a large amount of type I and II ROS after light irradiation, we further explored the application of Zr‐TPE‐MOF in the photocatalytic degradation of different types of organic dyes. Specifically, 0.3 mg mL^−1^ of Zr‐TPE‐MOF was added into aqueous solutions containing different organic dyes (MB, MO, RhB, 10 mg L^−1^). The color of the mixed solution containing Zr‐TPE‐MOF and organic dyes respectively becomes lighter, indicating that Zr‐TPE‐MOF can efficiently adsorb organic dyes, laying a foundation for its application in photocatalytic degradation of organic dyes. Therefore, we selected methylene blue (MB), methyl orange (MO), and rhodamine B (RhB) at a concentration of 10 mg L^−1^ to explore the degradation performance of Zr‐TPE‐MOF (0.3 mg mL^−1^) (**Figure** **6**a). A xenon lamp with a power density of 60 mW cm^−2^ was used to irradiate 30 mL of aqueous solutions containing different types of dyes. The solutions were taken out at 0, 5, 10, 15, 20, 30, and 40 min, respectively, and UV–visible (UV–vis) absorption spectra were tested. With the increase of light irradiation time, the intensity of characteristic absorption peaks at 665 nm (MB), 465 nm (MO), and 526 nm (RhB) gradually weakened, indicating that Zr‐TPE‐MOF has a good effect on the absorption of MB, MO, and RhB. It has a significant photodynamic degradation effect (Figure 6b–d). After 40 min of light irradiation, the degradation of high‐concentration MB, MO, and RhB solutions by Zr‐TPE‐MOF was 97.6%, 95.4%, and 75.3%, respectively. In addition, the degradation curves of the above dyes by Zr‐TPE‐MOF were following the first‐order kinetic equation ln(*C*_0_/*C*) = *κ*t. The rate constants κ of the photocatalytic degradation of MB, MO, and RhB by Zr‐TPE‐MOF were calculated to be 0.086, 0.067, and 0.027 min^−1^, respectively (Figure 6e–g). However, after the control aqueous solutions containing only MB, MO, and RhB were irradiated with light at the same power density for 40 min, their degradation rate constants *κ* were 0.001, 0.002, and 0.003 min^−1^, respectively (Figure 6; Figure S17, Supporting information). Obviously, after adding Zr‐TPE‐MOF into the aqueous solutions of MB, MO, and RhB, their photocatalytic degradation rates *κ* increased by 86, 67, and 27 times, respectively, indicating that Zr‐TPE‐MOF has an outstanding degradation effect on colored dyes. In addition, compared with most of the most common photocatalysts currently available (e.g., transition metal–organic framework Co‐MOF, Al‐MOF, covalent organic framework JUC‐598@FeSx, heterojunction structure ZnO/Bi_2_MoO_6_, etc.), Zr‐TPE‐MOF shows a faster and more efficient degradation effect of colored dyes, which is expected to open up new horizons for the development of new and more efficient photosensitizers (Figure 6h–j).^[^ ^12^, ^13^, ^15^, ^71^ ^]^ There are often mixtures of multiple organic dyes in daily sewage. Whether photosensitizers can degrade these mixed dyes in aqueous solution is crucial to their application prospects in dye wastewater purification. Zr‐TPE‐MOF with a concentration of 0.3 mg mL^−1^ was added to aqueous solutions containing mixed dyes CV (10 mg L^−1^) and MB (10 mg L^−1^), MO (10 mg L^−1^) and RhB (10 mg L^−1^), respectively. As the illumination time increased from 0 to 50 min, the absorbance of CV and MB decreased from 1.04 (a.u.) and 0.51 (a.u.) to 0.11 (a.u.) and 0.01 (a.u.), respectively, and the absorbance of MO and RhB decreased from 0.55 (a.u.) and 0.25 (a.u.) to 0.03 (a.u.) and 0.09 (a.u.), respectively (Figure S18, Supporting information). The degradation amounts of CV and MB in the mixed dyes by Zr‐TPE‐MOF were 89% and 98%, respectively, and the degradation amounts of MO and RhB mixed dyes were 94% and 88%, respectively. The control group without Zr‐TPE‐MOF showed no significant changes in the characteristic absorption peaks of organic dyes in aqueous solution after irradiation with the same power of light. The above results prove that Zr‐TPE‐MOF can still have an excellent photocatalytic degradation effect on mixed systems of various organic dyes, and can almost completely degrade the mixed dyes in an aqueous solution within 50 min.

To evaluate the stability of Zr‐TPE‐MOF after photodynamic degradation of organic dyes, the PXRD and SEM of Zr‐TPE‐MOF after photodynamic degradation of dyes were tested, respectively (Figure S19, Supporting information). The observed PXRD values of Zr‐TPE‐MOF after photodynamic degradation of organic dyes are consistent with the theoretical values of Zr‐TPE‐MOF (Figure S19a, Supporting information). In addition, the SEM results of Zr‐TPE‐MOF after photodynamic degradation of organic dyes showed that it still maintained a blocky morphology, with no obvious changes compared with the original Zr‐TPE‐MOF (Figure S19b, Supporting information). These results fully demonstrate that Zr‐TPE‐MOF still has excellent stability after the photodynamic degradation of organic dyes.

## 3 Conclusion

In conclusion, the flexible AIEgen (H_4_TPPE) with obvious molecular rotor structure was used as a linker to connect with the octahedral Zr_6_O_4_(OH)_8_(H_2_O)_4_ cluster‐based SBU to obtain the first smart luminescent MOF (Zr‐TPE‐MOF) with dynamic respiration driven by toxic vapors. Specifically, the introduction of a dynamic molecular rotor structure into the pores induces Zr‐TPE‐MOF to not only display thermofluorochromics behavior specific to high‐temperature regions but also to exhibit fast and highly sensitive intelligent light response behavior by inhaling trace amounts of CH_2_Cl_2_ or CH_3_COOH vapors through respiration. In addition, Zr‐TPE‐MOF with bright yellow‐green fluorescence dispersed in aqueous solution exhibits specific and highly sensitive smart fluorescence quenching for trace amounts of Cr_2_O_7_^2−^ ions. In addition, the portable test strips, after only a simple soaking in Zr‐TPE‐MOF, can identify extremely low concentrations of Cr_2_O_7_^2−^ ions in an aqueous solution with a high degree of naked‐eye visualization. The porous framework structure of Zr‐TPE‐MOF greatly increases the contact area and active sites with substrate molecules such as oxygen and H_2_O, and the organic‐inorganic hybrid characteristics effectively lead to a higher degree of charge separation, which together promotes the explosive generation of ROS under light irradiation conditions. The ROS storm induced by the Zr‐TPE‐MOF above has the properties of efficient photodynamic sterilization and photocatalytic degradation of organic dyes. To the best of our knowledge, this is the first time that a smart luminescent MOF with dynamic respiration driven by volatile toxic vapors has been discovered. This work not only opens new horizons for the construction of dynamic MOFs with smart light response but also promotes the development of artificial intelligence porous materials.

## 4 Experimental Section

### The Synthesis Method—Synthesis of Zr‐TPE‐MOF

ZrOCl_2_·8H_2_O (90 mg) and H_4_TPPE (30 mg) in a mixed solvent of DMF (9 mL) and HCOOH (6 mL) were charged in a vial. The mixture was ultrasound for 10 min and then was heated in a 120 °C oven for 24 h. After cooling down to room temperature, light‐green rod crystals. Elemental analysis theoretical value [C_60_H_54_O_16_N_2_Zr_3_]: C, 54.07%; H, 4.08%; N, 2.10%. Experimental value: C, 53.88%; H, 3.82%; N, 1.89%. Infrared spectrum data (IR, KBr pellet, cm^−1^): 3436 (s), 1627 (m), 1402 (m), 1108 (w), 791 (w), 641 (w).

### The Synthesis Method—Synthesis of Zr‐TPE‐MOF‐CH_2_Cl_2_

Zr‐TPE‐MOF‐CH_2_Cl_2_ was obtained by fumigating Zr‐TPE‐MOF with CH_2_Cl_2_ vapor (room temperature) for 12 h. Elemental analysis theoretical value (C_108.4_H_80.8_Cl_0.8_O_32_Zr_6_): C, 52.69%; H, 3.30%; experimental value: C, 51.52%; H, 3.05%.

### Statistical Analysis

Data between two groups was analyzed by an independent *t*‐test, and more than two groups were tested by one‐way ANOVA followed by a suitable post‐hoc analysis. For all tests, *p* < 0.05 was considered statistically significant. Data were expressed as the mean ± SD (standard deviation), *n*  =  3, unless other specified. All data were analyzed using GraphPad Prism 8.0 software.

## Conflict of Interest

The authors declare no conflict of interest.

## Supporting information



Supporting Information

## Data Availability

The data that support the findings of this study are available in the supplementary material of this article.
