# Supplementary material for: Respiration Drives Dynamic Metal–Organic Framework for Smart Photoresponse to Volatile Toxic Vapors and Their Photodynamic Sterilization
Source: Adv Sci (Weinh). 2025 May 20;12(27):2501824. doi: 10.1002/advs.202501824 (PMC12279190; doi:10.1002/advs.202501824)
Supplement: Supplementary file 1 — Supporting Information [file ADVS-12-2501824-s001.docx]

Supporting Information

Respiration Drives Dynamic Metal-organic Framework for Smart Photoresponse to Volatile Toxic Vapors and Their Photodynamic Sterilization

*Yun-Lan Li,^2^ Hai-Ling Wang,^1,^** *Ju-Fen Ai,^2^ Guan-Huang Zhang,^2^ Hua-Hong Zou,^2^ Fu-Pei Liang,^2^ and Zhong-Hong Zhu^1,^**

*^1^ School of Chemistry and Chemical Engineering, Guangxi Key Laboratory of Electrochemical Energy Materials, Guangxi University, Nanning, 530004, P. R. China*

*^2^ School of Chemistry and Pharmaceutical Sciences, State Key Laboratory for Chemistry and Molecular Engineering of Medicinal Resources, Guangxi Normal University, Guilin 541004, P. R. China*

*E-mail (Corresponding author): whling@gxu.edu.cn (H.-L. Wang), zzhong@gxu.edu.cn (Z.-H. Zhu).

**Table of Contents:**

| Supporting Tables | |
| --- | --- |
| Table S1 | Crystallographic data of Zr-TPE-MOF and Zr-TPE-MOF-CH_2_Cl_2_. |
| Table S2 | Selected bond lengths (Å) and angles (°) of Zr-TPE-MOF and Zr-TPE-MOF-CH_2_Cl_2_. |
| Supporting Figures | |
| Figure S1 | SEM, TEM, and EDS of Zr-TPE-MOF. |
| Figure S2 | FT-IR spectra of Zr-TPE-MOF. |
| Figure S3 | Thermogravimetric curves of Zr-TPE-MOF. |
| Figure S4 | Powder X-ray diffraction patterns (PXRD) of Zr-TPE-MOF. |
| Figure S5 | PXRD patterns of Zr-TPE-MOF immersed in different solvents (a) and pH aqueous solutions (pH = 3, 5, 7, 9, and 11) (b). |
| Figure S6 | Three-dimensional fluorescence spectrum of Zr-TPE-MOF in DMSO : H_2_O (1 : 99). |
| Figure S7 | Fluorescence lifetime (a-c) and quantum yield (d-f) of Zr-TPE-MOF, Zr-TPE-MOF-CH_3_COOH, and Zr-TPE-MOF-CH_2_Cl_2_. |
| Figure S8 | PXRD results of Zr-TPE-MOF at 320 K ~ 440 K. |
| Figure S9 | Linear fitting plot of the emission intensity of Zr-TPE-MOF versus the vapor concentration of CH_2_Cl_2_ (a) and CH_3_COOH (b). |
| Figure S10 | Solid UV-Vis of Zr-TPE-MOF, Zr-TPE-MOF-CH_3_COOH, and Zr-TPE-MOF-CH_2_Cl_2_. |
| Figure S11 | Dihedral angles on the ligand arms in Zr-TPE-MOF (a) and Zr-TPE-MOF-CH_2_Cl_2_ (b). |
| Figure S12 | UV-Vis of Zr-TPE-MOF-Cr_2_O_7_^2-^. |
| Figure S13 | (a and d) Fluorescence spectra of aqueous solutions containing only DHR 123 and HPF (20 μM) under different illumination times (60 mW·cm^−2^); (b and e) Fluorescence spectra of aqueous solutions containing DHR 123 and HPF (20 μM) with a concentration of 10 μg·mL^−1^ Zr-TPE-MOF under different illumination times (60 mW·cm^−2^); (c and f) ·O_2_^−^ and ·OH generation capacities of Zr-TPE-MOF using DHR 123 and HPF as indicators, respectively. |
| Figure S14 | (a) Absorption spectra change of aqueous solution containing only ABDA (0.1 mM) after light irradiation (60 mW·cm^−2^) for different time periods; Absorption spectra change of aqueous solution containing ABDA (0.1 mM) containing Zr-TPE-MOF (b) with increasing light irradiation (60 mW·cm^−2^) time; ^1^O_2_ generation capacities and rates of Zr-TPE-MOF using ABDA as the indicator. |
| Figure S15 | (a-d) The size of the inhibition zone of Zr-TPE-MOF against *E. coli* and *S. aureus* under different illumination time and power conditions; (e) Photodynamic bactericidal effect of Zr-TPE-MOF on *E. coli* and *S. aureus* under different illumination time and power conditions (Repeat 3 times, *n* = 3). (Note: Data represent mean ±SD. n = 3, (a) ****p* < 0.0009 and No significant difference, *p* = 0.0891; (b) ***p* < 0.0025 and No significant difference, p = 0.08697; (c) *****p* < 0.0001 and No significant difference, *p* = 0.0704; (d) *****p* < 0.0001 and ***p* < 0.0070.) |
| Figure S16 | Photos of the antibacterial effect of Zr-TPE-MOF (3 mg·mL^−1^) on *E. coli* and *S.* *aureus* under dark (blank) and light conditions (60 mW·cm^−2^, 5 min) (Repeat 3 times, n = 3). |
| Figure S17 | UV–Visible absorption spectra of organic dye (MB = MO = RhB = 10 mg·L^−1^) aqueous solutions (pH = 7.0) under different illumination times (60 mW·cm^-2^). |
| Figure S18 | (a and b) UV-visible absorption spectra of mixed dye aqueous solutions of CV (10 mg·L^−1^) and MB (10 mg·L^−1^) in the absence and presence of Zr-TPE-MOF (0.3 mg·mL^−1^) as a function of light irradiation time; (c and d) UV-visible absorption spectra of mixed dye aqueous solutions of MO (10 mg·L^−1^) and RhB (10 mg·L^−1^) in the absence and presence of Zr-TPE-MOF (0.3 mg·mL^−1^) as a function of light irradiation time (Repeat 3 times, *n* = 3); Degradation curves (e-h) and degradation kinetic fitting diagrams (i-l) of the above Zr-TPE-MOF (0.3 mg·mL^−1^) degradation of the mixed dye system (Repeat 3 times, *n* = 3). |
| Figure S19 | (a) PXRD of Zr-TPE-MOF after photocatalytic degradation of organic dye aqueous; (b) SEM of Zr-TPE-MOF after photocatalytic degradation of organic dye aqueous. |

**Experimental Section**

**Materials and measurements.**

All reagents were obtained from commercial sources and used without further purification. Elemental analyses for C, H, and N were performed on a varia MICRO cube. Fourier transform infrared (FT-IR) spectra were recorded by transmission through KBr pellets containing *ca.* 0.5% of the complexes using a PE Spectrum FT-IR spectrometer (400–4,000 cm^−1^). Thermogravimetric analyses (TGA) were conducted in a flow of nitrogen at a heating rate of 5 °C/min using a NETZSCH TG 209 *F*^3^. UV-Vis absorption spectra were recorded on a Cary 100 UV-Vis spectrophotometer (Agilent), and fluorescence spectra were recorded on a Cary Eclipse fluorescence spectrophotometer (Agilent) and Quanta Master 8000 (HORIBA CANADA). The scanning electron microscopy (SEM) images were recorded using FEI Quanta 200 Field Emission Environmental Scanning Electron Microscopy. Powder X-ray diffraction (PXRD) spectra were recorded on either a D8 Advance (Bruker) diffractometer at 293 K (Mo-*K*α). The samples were prepared by crushing crystals and the powder was placed on a grooved aluminum plate. Diffraction patterns were recorded from 5º to 55º at a rate of 5º min^−1^. HRTEM, lattice fringes, electron diffraction spots, and EDS of Zr-TPE-MOF were all tested by ThermoFisher Scientific Talos F200S.

**Single-crystal X-ray crystallography.**

Diffraction data for the complex were collected on a Bruker SMART CCD diffractometer (Cu-Kα radiation and *λ* = 1.54 Å) in *Φ* and *ω* scan modes. The structures were solved by direct methods, followed by difference Fourier syntheses, and then refined by full-matrix least-squares techniques on *F*^2^ using *SHELXL*.^[1]^ All other non-hydrogen atoms were refined with anisotropic thermal parameters. Hydrogen atoms were placed at calculated positions and isotopically refined using a riding model. Table S1 summarizes X-ray crystallographic data and refinement details for the complexes. The CCDC reference numbers are 2419611 (Zr-TPE-MOF) and 2419612 (Zr-TPE-MOF-CH_2_Cl_2_).

**Photodynamic sterilization experiment.**

Specifically, the strains were recovered after 24 h using a 37 °C biochemical incubator, then inoculated into LB liquid culture medium for culture, and finally diluted with 0.9% (w/v) saline to prepare suspensions of *E. coli* and *S. aureus* as 0.5 McMahon turbidity standards (approximately 2×10^8^ CFU mL^−1^) for use. Solutions of Zr-TPE-MOF with a concentration of 3 mg·mL^−1^ were prepared separately for use. The drug-susceptibility paper sheets were immersed in the above solutions for 30 s, then quickly taken out and dried. The light irradiation experimental groups were irradiated with a xenon lamp with a low power density of 60 mW/cm^2^ for 5 min and then transferred to a biochemical incubator for 24 h.

The culture and concentration of the bacteria were the same as those in the above experiment. 100 μL of *E. coli* and *S. aureus* liquid were accurately pipetted and evenly spread on the LA medium. Medical masks cut into 1×1 cm pieces were immersed in a solution containing 3 mg·mL^−1^ of Zr-TPE-MOF. After 30 s, they were taken out and added to the solid culture medium coated with the bacterial solution. The cells in the illumination experiment group were then placed under a 60 mW·cm^−2^ X-lamp for 5 min and then cultured in a biochemical incubator for 24 h.

**The concentration of CH_3_COOH and CH_2_Cl_2_ vapor.**

According to the Ideal Gas Law *pV* = *nRT* (*p* is the saturated vapor pressure of CH_3_COOH or CH_2_Cl_2_ vapor (Pa), *V* is the CH_3_COOH or CH_2_Cl_2_ vapor volume (m^3^), *T* is the temperature (K), *n* is the amount of substance of CH_3_COOH or CH_2_Cl_2_ vapor (mol), *R* is the molar gas constant (*J*/(mol·K))), the concentration CH_3_COOH or CH_2_Cl_2_ vapor above the surface of CH_3_COOH or CH_2_Cl_2_ solution in the small sealed glass bottle can be directed calculated from the vapor pressure of the hydrochloric acid solution, using *c* = *p* / RT (c is the concentration of CH_3_COOH or CH_2_Cl_2_ vapor).

**Reactive oxygen species (ROS) detection**

**Total ROS detection:**

2',7'-Dichlorodihydrofluorescein diacetate (DCFH-DA) was activated with NaOH (0.01 M) to obtain DCFH, which was used as a ROS indicator.^[2-5]^ 20 μL of Zr-TPE-MOF solutions (1 mM) were added to 1980 μL of DCFH (50 μM) aqueous solution. The fluorescence spectra of the above solutions after irradiation with a xenon lamp (60 mW·cm^−2^) for different time periods were monitored using a fluorescence spectrometer (*λ_e_*_x_ = 480 nm).

**Superoxide anion radical (·O_2_^-^) detection:**

Dihydrorhodamine 123 (DHR 123) was used as an indicator of ·O_2_^-^. The specific steps were as follows: 10 μL of photosensitizer stock solution (Zr-TPE-MOF, 1 mg·mL^−1^) was added to an aqueous solution containing 20 μM DHR 123 to a final concentration of 10 μg·mL^−1^. After the above solution was irradiated with white light emitted by a 60 mW·cm^−2^ xenon lamp at different times, the changes in the indicator fluorescence signal were monitored by a fluorescence spectrometer. The excitation wavelength was 480 nm, and the fluorescence intensity of DHR 123 at 525 nm was recorded, indicating the generation of superoxide anion radicals.

**Hydroxyl radical (·OH) detection:**

Hydroxyphenylfluorescein (HPF) was used to monitor the generation of ·OH. The specific steps are as follows: In an aqueous solution containing 10 μM HPF, 10 μL of photosensitizer stock solution (Zr-TPE-MOF, 1 mg·mL^−1^) was added to a final concentration of 10 μg·mL^−1^. After the above solution was irradiated with white light from a 60 mW·cm^−2^ xenon lamp at different times, the changes in the indicator fluorescence signal were monitored by a fluorescence spectrometer. The excitation wavelength was 480 nm, and the fluorescence intensity of HPF at 515 nm was recorded to indicate the generation of ·OH.

**Singlet oxygen (^1^O_2_) detection:**

ABDA detection of singlet oxygen: 9,10-Anthracenediyl-di(methylene)dimalonic acid (ABDA) was used to measure the generation of ^1^O_2_. The specific steps are as follows: the photosensitizer sample solution (DMSO; Zr-TPE-MOF; 10 μL), with a stock concentration of 1 mg·mL^−1^, was added to a 20 μM ABDA aqueous solution. After the above solution was irradiated with white light from a 60 mW·cm^−2^ xenon lamp for different times, the change in the absorption signal of the indicator ABDA at 380 nm was monitored by a UV-Vis spectrophotometer to monitor the generation of ^1^O_2_.

**Photocatalytic dye degradation**

Dyes (MB, MO, and RhB)：

0.3 mg·mL^−1^ Zr-TPE-MOF were added to 10 mg·L^−1^ dye aqueous solution, respectively. The solutions were irradiated with a xenon lamp (60 mW·cm^−2^) for different time periods. The reaction solutions were taken out at set time points (0, 5, 10, 15, 20, 30, and 40 min), the insoluble matter in the solution was removed, and the absorbance of the dye was measured with a UV-Vis absorption meter.

Mixed dyes (MB and CV, MO and RhB)

0.3 mg·mL^−1^ Zr-TPE-MOF was added to aqueous solutions of mixed dyes MB (10 mg·L^−1^) and CV (10 mg·L^−1^) as well as MO (10 mg·L^−1^) and RhB (10 mg·L^−1^), respectively. The mixture was irradiated with a xenon lamp (60 mW·cm^−2^) for different times. The reaction solutions were taken out at set time points (0, 5, 10, 15, 20, 30, 40, 50 min), the insoluble matter in the solution was removed, and the absorbance of the dye was measured using a UV-visible absorption spectrometer.

**Table S1.** Crystallographic data of Zr-TPE-MOF and Zr-TPE-MOF-CH_2_Cl_2_.

|  | Zr-TPE-MOF | Zr-TPE-MOF-CH_2_Cl_2_ |
| --- | --- | --- |
| Formula | C_60_H_54_O_16_N_2_Zr_3_ | C_108.4_H_80.8_Cl_0.8_O_32_Zr_6_ |
| Formula weight | 1332.71 | 2471.01 |
| *T*, K | 100.00(11) | 100.00(11) |
| Crystal system | orthorhombic | orthorhombic |
| Space group | *Fmmm* | *Cmme* |
| *a*, Å | 15.95380(10) | 20.7315(3) |
| *b*, Å | 40.8781(3) | 42.4550(6) |
| *c*, Å | 29.1688(4) | 18.0589(3) |
| *α*, ^o^ | 90.00 | 90.00 |
| *β*, ^o^ | 90.00 | 90.00 |
| *γ*, ^o^ | 90.00 | 90.00 |
| *V*, Å^3^ | 19022.8(3) | 15894.6(4) |
| *Z* | 8 | 4 |
| *D*_c_, g·cm^-3^ | 0.931 | 1.033 |
| *μ*, mm^-1^ | 3.009 | 3.682 |
| *F*(000) | 5408.0 | 4963.0 |
| 2*θ* range for data collection/° | 6.06 to 154.214 | 6.426 to 136.496 |
| Reflns coll.  Unique reflns  *R*_int_ | 26110  5177  0.0340 | 30279  7539  0.0878 |
| *R*_1_^a^ (*I* > 2σ(*I*)) | 0.0565 | 0.1022 |
| *wR*_2_^b^ (all data) | 0.1707 | 0.2826 |
| GOF | 1.057 | 1.033 |

^a^*R*_1_=Σ||*F*_o_|-|*F*_c_||/Σ|*F*_o_|, ^b^w*R*_2_ = [Σw(*F*_o_^2^-*F*_c_^2^)^2^/Σw(*F*_o_^2^)^2^]^1/2^

**Table S2.** Selected bond lengths (Å) and angles (°) of Zr-TPE-MOF and Zr-TPE-MOF-CH_2_Cl_2_.

| **Bond lengths of Zr-TPE-MOF (Å)** | | | | | |
| --- | --- | --- | --- | --- | --- |
|  | | | | | |
| Zr1-O1^iv^ | 2.195(2) | Zr1-O3^iv^ | 2.171(4) | Zr2-O3 | 2.118(3) |
| Zr1-O1^v^ | 2.195(2) | Zr1-O3 | 2.171(4) | Zr2-O6 | 2.174(5) |
| Zr1-O1^iii^ | 2.195(2) | Zr2-O2^i^ | 2.254(3) | Zr2-O5 | 2.200(5) |
| Zr1-O1 | 2.195(2) | Zr2-O2^iv^ | 2.254(3) | Zr2-O4^ii^ | 2.134(3) |
| Zr1-O4 | 2.171(4) | Zr2-O4 | 2.134(3) | Zr2-O3^vi^ | 2.118(3) |
| Zr1-O4^iii^ | 2.171(4) |  |  |  |  |
| **Bond angles of Zr-TPE-MOF (°)** | | | | | |
| O1-Zr1-O1^iii^ | 74.15(14) | O4^iii^-Zr1-O1^v^ | 78.98(14) | O4-Zr2-O2^iv^ | 75.47(15) |
| O1^v^-Zr1-O1^iii^ | 117.40(13) | O3-Zr1-O4^iii^ | 70.01(11) | O4^ii^-Zr2-O2^i^ | 75.48(15) |
| O1^iv^-Zr1-O1^iii^ | 74.53(14) | O3-Zr1-O4 | 70.02(11) | O4^ii^-Zr2-O2^iv^ | 139.26(14) |
| O1-Zr1-O1^v^ | 74.53(14) | O3-Zr1-O3^iv^ | 107.6(3) | O4-Zr2-O2^i^ | 139.26(14) |
| O1-Zr1-O1^iv^ | 117.40(13) | O4^iii^-Zr1-O1^iv^ | 142.40(7) | O4^ii^-Zr2-O4 | 68.2(2) |
| O1^iv^-Zr1-O1^v^ | 74.15(14) | O4^iii^-Zr1-O1 | 78.98(14) | O4^ii^-Zr2-O6 | 145.90(11) |
| O4-Zr1-O1^iii^ | 78.98(14) | O3-Zr1-O1^iii^ | 142.70(7) | O4-Zr2-O6 | 145.90(11) |
| O4-Zr1-O1^iv^ | 78.98(14) | O3^iv^-Zr1-O4^iii^ | 70.01(11) | O4^ii^-Zr2-O5 | 86.9(2) |
| O4^iii^-Zr1-O4 | 109.3(3) | O3^iv^-Zr1-O4 | 70.01(11) | O4-Zr2-O5 | 86.9(2) |
| O3-Zr1-O1 | 142.70(7) | O3^vi^-Zr2-O2^iv^ | 139.25(14) | O2^i^-Zr2-O2^iv^ | 128.30(15) |
| O3^iv^-Zr1-O1^iv^ | 142.70(7) | O3-Zr2-O2^iv^ | 76.32(16) | O3-Zr2-O4^ii^ | 107.8(2) |
| O3^iv^-Zr1-O1^v^ | 142.70(7) | O3-Zr2-O2^i^ | 139.25(14) | O3-Zr2-O4 | 71.71(17) |
| O3-Zr1-O1^v^ | 79.53(14) | O3^vi^-Zr2-O2^i^ | 76.33(16) | O3^vi^-Zr2-O4^ii^ | 71.71(17) |
| O3^iv^-Zr1-O1^iii^ | 79.54(15) | O5-Zr2-O2^iv^ | 73.15(11) | O3^vi^-Zr2-O4 | 107.8(2) |
| O3-Zr1-O1^iv^ | 79.53(14) | O6-Zr2-O2^i^ | 72.62(11) | O3^vi^-Zr2-O3 | 67.0(2) |
| O3^iv^-Zr1-O1 | 79.54(15) | O6-Zr2-O2^iv^ | 72.62(11) | O3-Zr2-O6 | 89.2(2) |
| O4-Zr1-O1^v^ | 142.40(7) | O6-Zr2-O5 | 95.1(2) | O3^vi^-Zr2-O6 | 89.2(2) |
| O4^iii^-Zr1-O1^iii^ | 142.40(7) | O3^vi^-Zr2-O5 | 146.25(11) | O3-Zr2-O5 | 146.25(11) |
| O4-Zr1-O1 | 142.40(7) | O5-Zr2-O2^i^ | 73.15(11) |  |  |
| **Bond lengths of Zr-TPE-MOF-CH_2_Cl_2_ (Å)** | | | | | |
| Zr1-O1 | 2.188(6) | Zr2-O8 | 2.117(11) | Zr3-O2^i^ | 2.267(6) |
| Zr1-O1^ii^ | 2.188(6) | Zr2-O7 | 2.108(5) | Zr3-O2^ii^ | 2.267(6) |
| Zr1-O4^iii^ | 2.194(6) | Zr2-O7^i^ | 2.108(5) | Zr3-O10 | 2.166(11) |
| Zr1-O4^iv^ | 2.194(6) | Zr2-O6 | 2.128(7) | Zr3-O6^v^ | 2.126(6) |
| Zr1-O7 | 2.172(8) | Zr2-O6^v^ | 2.128(7) | Zr3-O6 | 2.126(6) |
| Zr1-O6 | 2.184(7) | Zr2-O3^iii^ | 2.272(6) | Zr3-O5 | 2.133(5) |
| Zr1-O6^ii^ | 2.184(7) | Zr2-O3^vi^ | 2.272(6) | Zr3-O5^i^ | 2.133(5) |
| Zr1-O5 | 2.181(9) | Zr2-O9 | 2.181(11) | Zr3-O11 | 2.252(9) |
| **Bond angles of Zr-TPE-MOF-CH_2_Cl_2_ (°)** | | | | | |
| O1^ii^-Zr1-O1 | 74.5(4) | O8-Zr2-O3^vi^ | 72.2(2) | O6-Zr2-O6^v^ | 67.2(3) |
| O1^ii^-Zr1-O4^iv^ | 117.9(2) | O8-Zr2-O3^iii^ | 72.2(2) | O6^v^-Zr2-O3^vi^ | 76.6(3) |
| O1^ii^-Zr1-O4^iii^ | 74.9(2) | O8-Zr2-O9 | 92.9(5) | O6-Zr2-O3^iii^ | 76.6(3) |
| O1-Zr1-O4^iv^ | 74.9(2) | O7^i^-Zr2-O8 | 88.4(4) | O6^v^-Zr2-O3^iii^ | 139.7(3) |
| O1-Zr1-O4^iii^ | 117.9(2) | O7-Zr2-O8 | 88.4(4) | O6-Zr2-O3^vi^ | 139.7(3) |
| O7-Zr1-O1^ii^ | 142.59(18) | O7-Zr2-O7^i^ | 66.8(4) | O6-Zr2-O9 | 89.5(4) |
| O7-Zr1-O1 | 142.59(18) | O7^i^-Zr2-O6 | 107.4(4) | O6^v^-Zr2-O9 | 89.5(4) |
| O7-Zr1-O4^iii^ | 79.5(3) | O7^i^-Zr2-O6^v^ | 71.9(3) | O3^vi^-Zr2-O3^iii^ | 128.3(3) |
| O7-Zr1-O4^iv^ | 79.5(3) | O7-Zr2-O6 | 71.9(3) | O9-Zr2-O3^iii^ | 72.9(2) |
| O7-Zr1-O6^ii^ | 69.7(2) | O7-Zr2-O6^v^ | 107.4(4) | O9-Zr2-O3^vi^ | 72.9(2) |
| O7-Zr1-O6 | 69.7(2) | O7-Zr2-O3^iii^ | 75.8(3) | O10-Zr3-O2^ii^ | 74.2(2) |
| O7-Zr1-O5 | 108.2(4) | O7^i^-Zr2-O3^vi^ | 75.8(3) | O10-Zr3-O2^i^ | 74.2(2) |
| O6^ii^-Zr1-O1^ii^ | 142.4(2) | O7^i^-Zr2-O3^iii^ | 138.3(3) | O10-Zr3-O11 | 95.0(5) |
| O6-Zr1-O1^ii^ | 79.0(3) | O7-Zr2-O3^vi^ | 138.3(3) | O2^ii^-Zr3-O2^i^ | 127.9(3) |
| O6-Zr1-O1 | 142.4(2) | O7^i^-Zr2-O9 | 146.6(2) | O6^v^-Zr3-O5^i^ | 72.3(3) |
| O6^ii^-Zr1-O1 | 79.0(3) | O7-Zr2-O9 | 146.6(2) | O6-Zr3-O11 | 146.18(18) |
| O6^ii^-Zr1-O4^iv^ | 79.2(3) | O11-Zr3-O2^ii^ | 71.4(2) | O6^v^-Zr3-O11 | 146.18(18) |
| O6-Zr1-O4^iii^ | 79.2(3) | O11-Zr3-O2^i^ | 71.4(2) | O6-Zr3-O2^i^ | 141.2(3) |
| O6^ii^-Zr1-O4^iii^ | 142.2(2) | O5-Zr3-O2^ii^ | 74.3(3) | O6^v^-Zr3-O2^ii^ | 141.2(3) |
| O6^ii^-Zr1-O6 | 108.6(4) | O5^i^-Zr3-O2^i^ | 74.3(3) | O6-Zr3-O2^ii^ | 77.5(3) |
| O5-Zr1-O1 | 78.7(3) | O5^i^-Zr3-O2^ii^ | 137.0(3) | O6^v^-Zr3-O2^i^ | 77.5(3) |
| O5-Zr1-O1^ii^ | 78.7(3) | O5-Zr3-O2^i^ | 137.0(3) | O6^v^-Zr3-O10 | 89.0(4) |
| O5-Zr1-O4^iv^ | 142.7(2) | O5^i^-Zr3-O10 | 146.1(2) | O6-Zr3-O10 | 89.0(4) |
| O5-Zr1-O4^iii^ | 142.7(2) | O5-Zr3-O10 | 146.1(2) | O6-Zr3-O6^v^ | 67.3(4) |
| O5-Zr1-O6^ii^ | 70.3(2) | O5-Zr3-O5^i^ | 67.8(4) | O6-Zr3-O5^i^ | 108.3(4) |
| O5-Zr1-O6 | 70.3(2) | O5-Zr3-O11 | 86.5(4) | O6-Zr3-O5 | 72.3(3) |
| O8-Zr2-O6 | 146.34(17) | O5^i^-Zr3-O11 | 86.5(4) | O6^v^-Zr3-O5 | 108.3(4) |
| O8-Zr2-O6^v^ | 146.34(17) |  |  |  |  |

Symmetry codes of Zr-TPE-MOF: (i) -*x*+1, -*y*+1, -*z*+1; (ii) -*x*+1, -*y*+1, *z*; (iii) *x*, *y*, -*z*+1; (iv) -*x*+1, *y*, -*z*+1; (v) -*x*+1, *y*, *z*; (vi) *x*, -*y*+1, *z*; (vii) -*x*+2, -*y*+3/2, -*z*+3/2; (viii) *x*, -*y*+3/2, -*z*+3/2.; and Symmetry codes of Zr-TPE-MOF-CH_2_Cl_2_: (i) -*x*+1, -*y*+1, -*z*+1; (ii) -*x*+1, -*y*+1, *z*; (iii) *x*, *y*, -*z*+1; (iv) -*x*+1, *y*, -*z*+1; (v) -*x*+1, *y*, *z*; (vi) *x*, -*y*+1, *z*; (vii) -*x*+2, -*y*+3/2, -*z*+3/2; (viii) *x*, -*y*+3/2, -*z*+3/2.


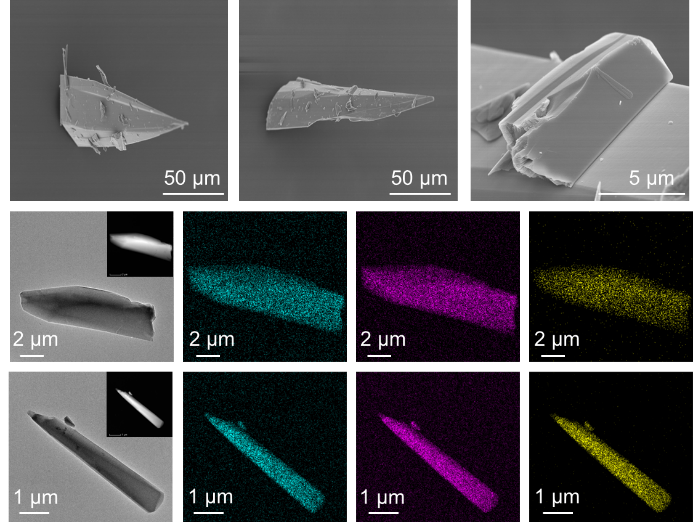


**Figure S1.** SEM, TEM, and EDS of Zr-TPE-MOF.

**
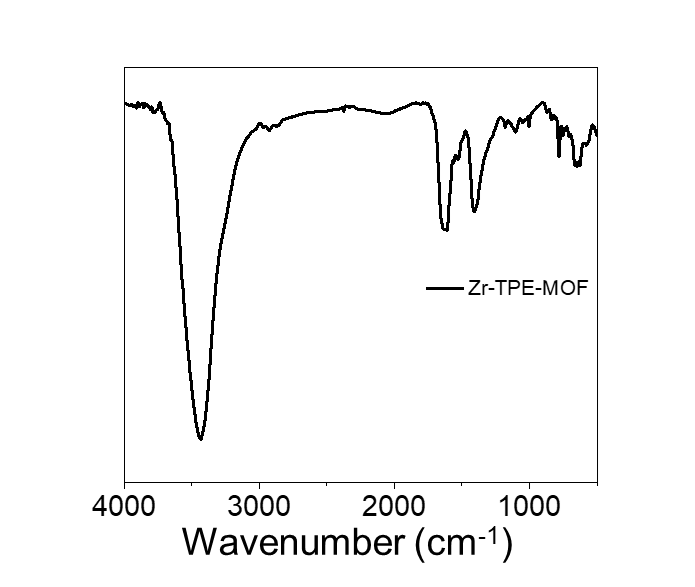
**

**Figure S2.** FT-IR spectra of Zr-TPE-MOF.

**
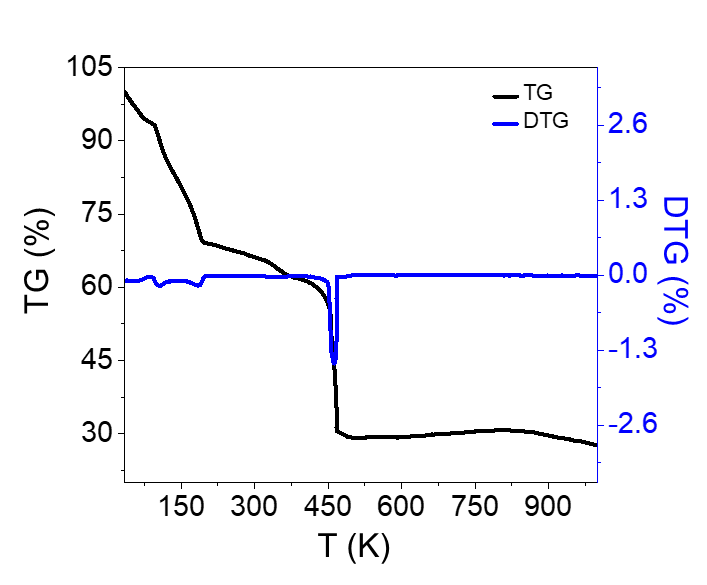
**

**Figure S3.** Thermogravimetric curves of Zr-TPE-MOF.

**
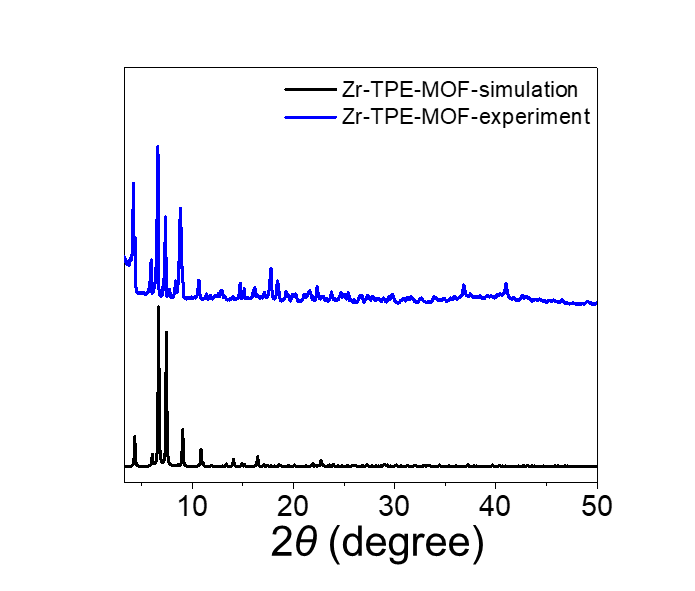
**

**Figure S4.** Powder X-ray diffraction patterns (PXRD) of Zr-TPE-MOF.

**
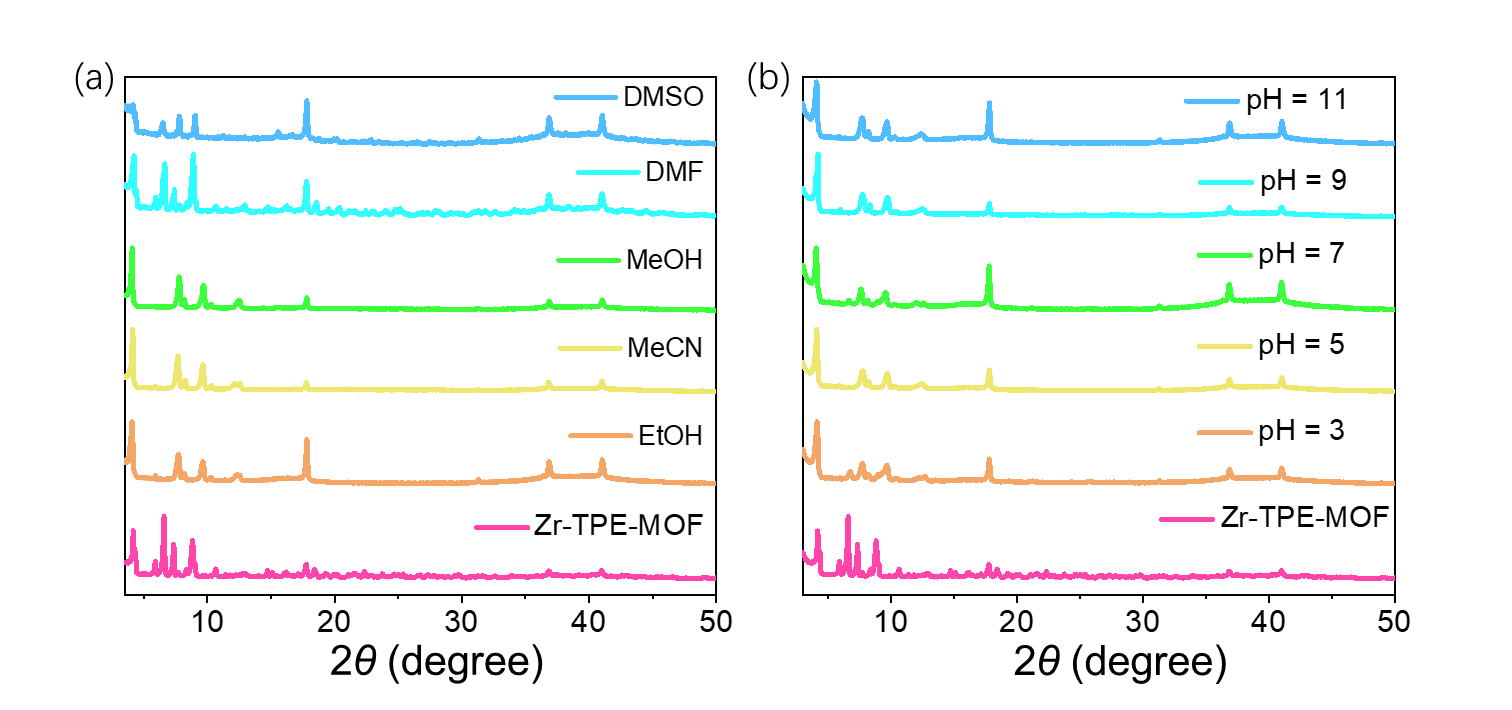
**

**Figure S5.** PXRD patterns of Zr-TPE-MOF immersed in different solvents (a) and pH aqueous solutions (pH = 3, 5, 7, 9, and 11) (b).


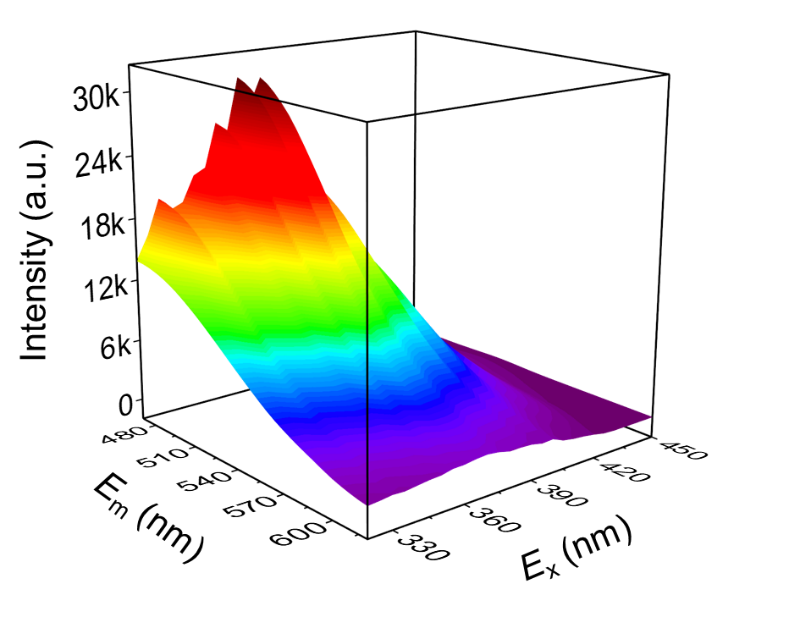


**Figure S6.** Three-dimensional fluorescence spectrum of Zr-TPE-MOF in DMSO : H_2_O (1 : 99).


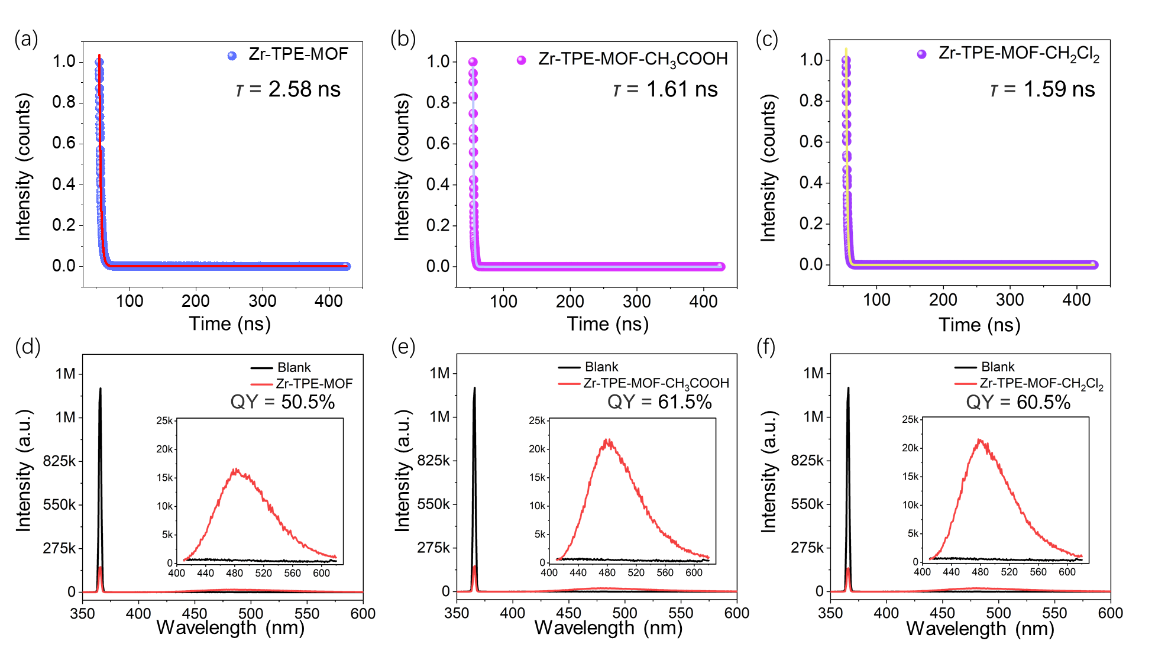


**Figure S7.** (a) Fluorescence lifetime (a-c) and quantum yield (d-f) of Zr-TPE-MOF, Zr-TPE-MOF-CH_3_COOH, and Zr-TPE-MOF-CH_2_Cl_2_.


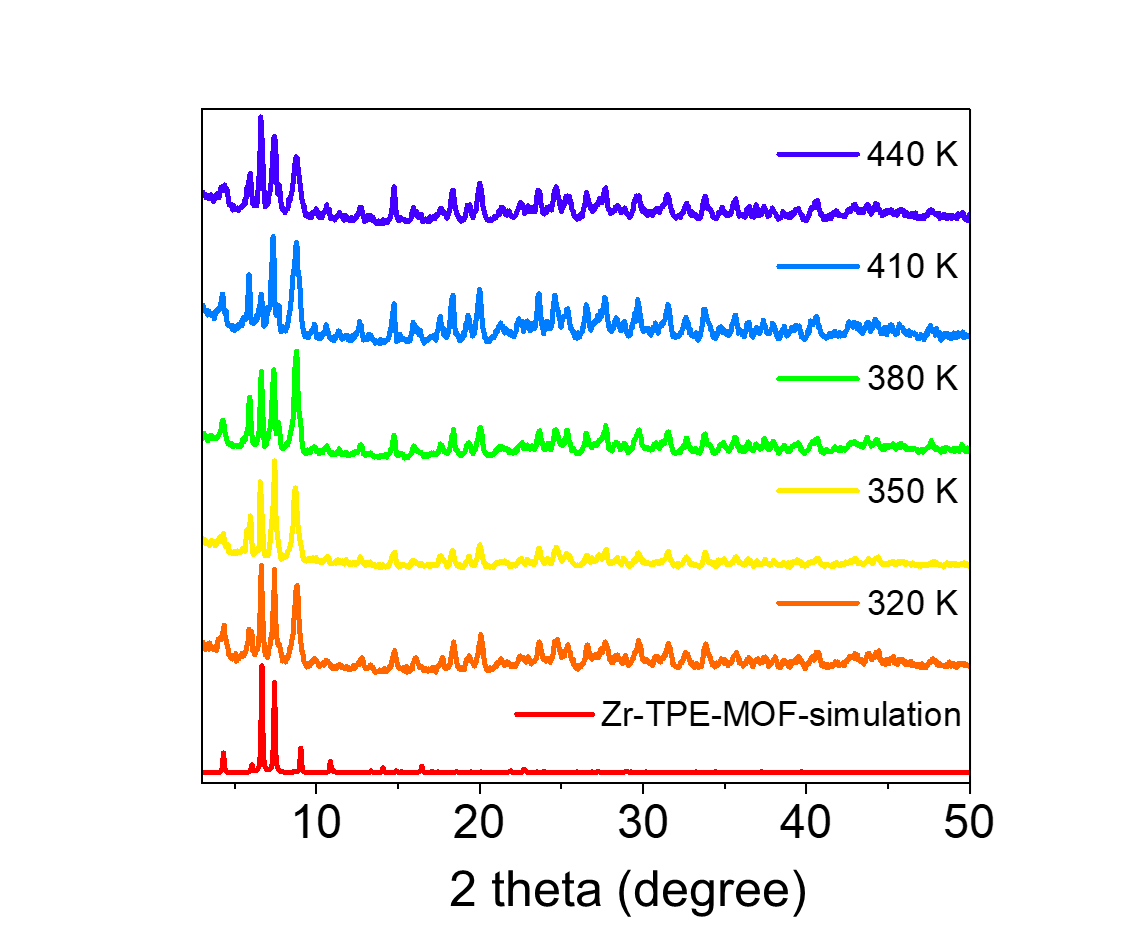


**Figure S8.** PXRD results of Zr-TPE-MOF at 320 K ~ 440 K.


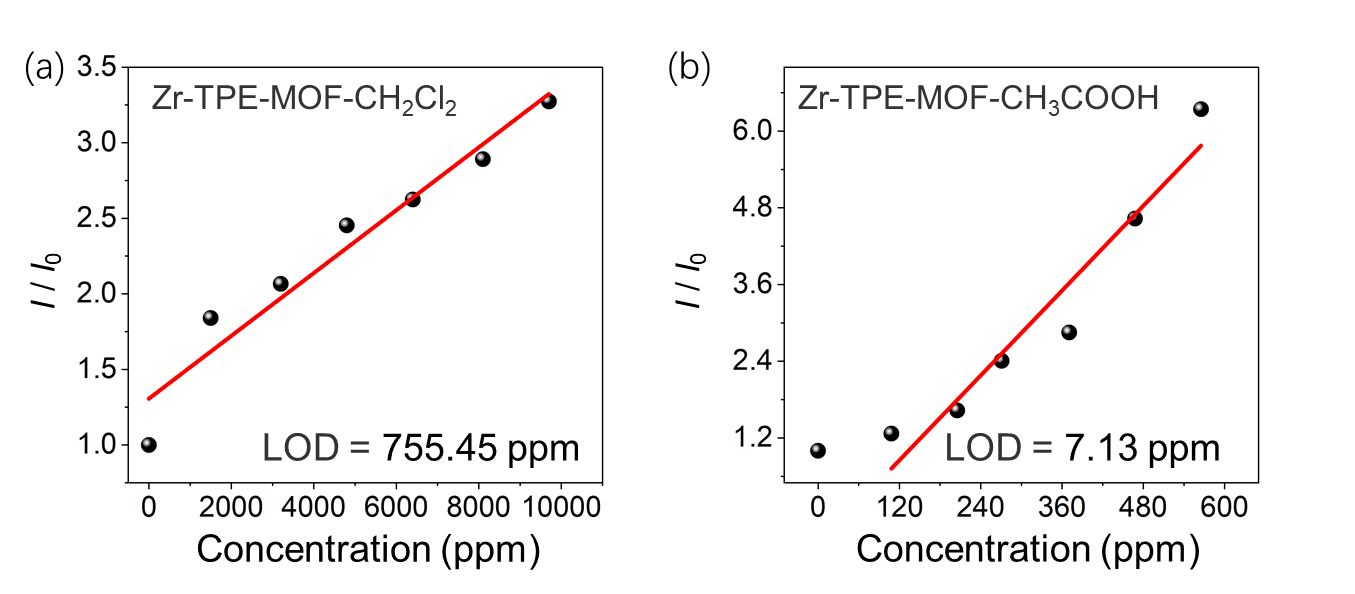


**Figure S9.** Linear fitting plot of the emission intensity of Zr-TPE-MOF versus the vapor concentration of CH_2_Cl_2_ (a) and CH_3_COOH (b).


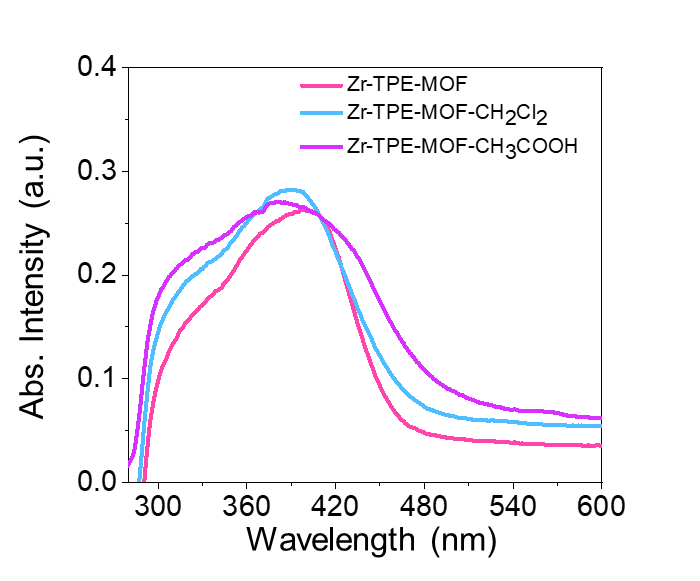


**Figure S10.** Solid UV-Vis of Zr-TPE-MOF, Zr-TPE-MOF-CH_3_COOH, and Zr-TPE-MOF-CH_2_Cl_2_.

**
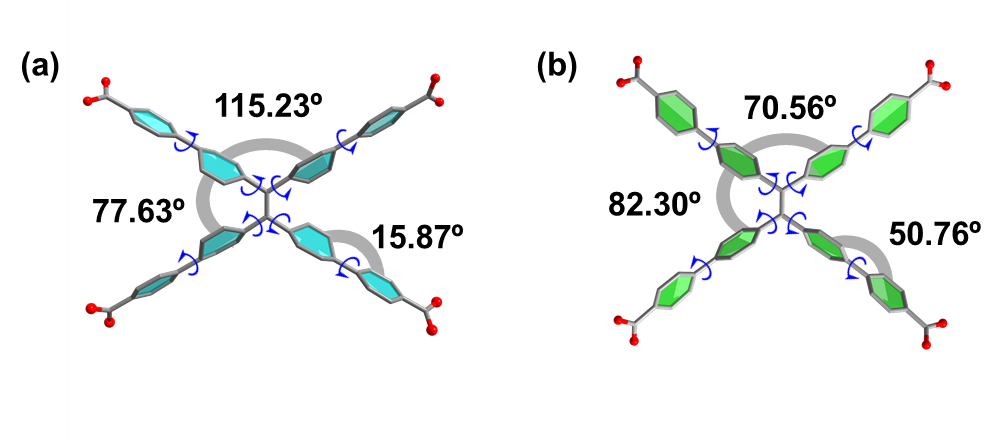
**

**Figure S11.** Dihedral angles on the ligand arms in Zr-TPE-MOF (a) and Zr-TPE-MOF-CH_2_Cl_2_ (b).


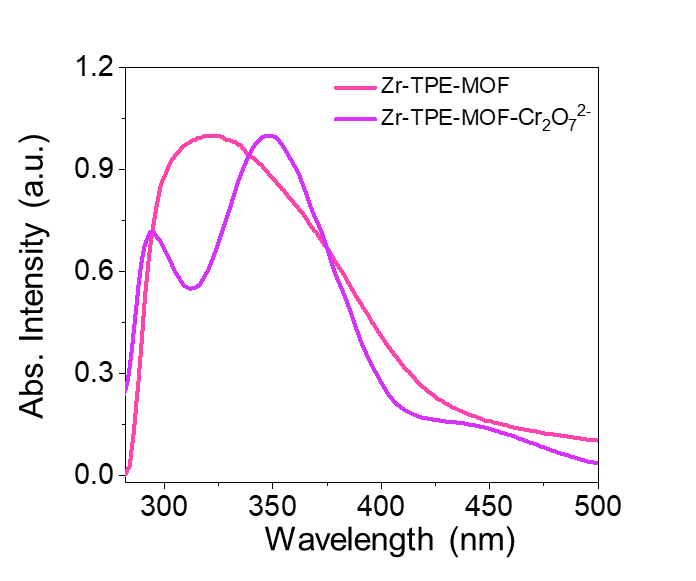


**Figure S12.** UV-Vis of Zr-TPE-MOF-Cr_2_O_7_^2-^.


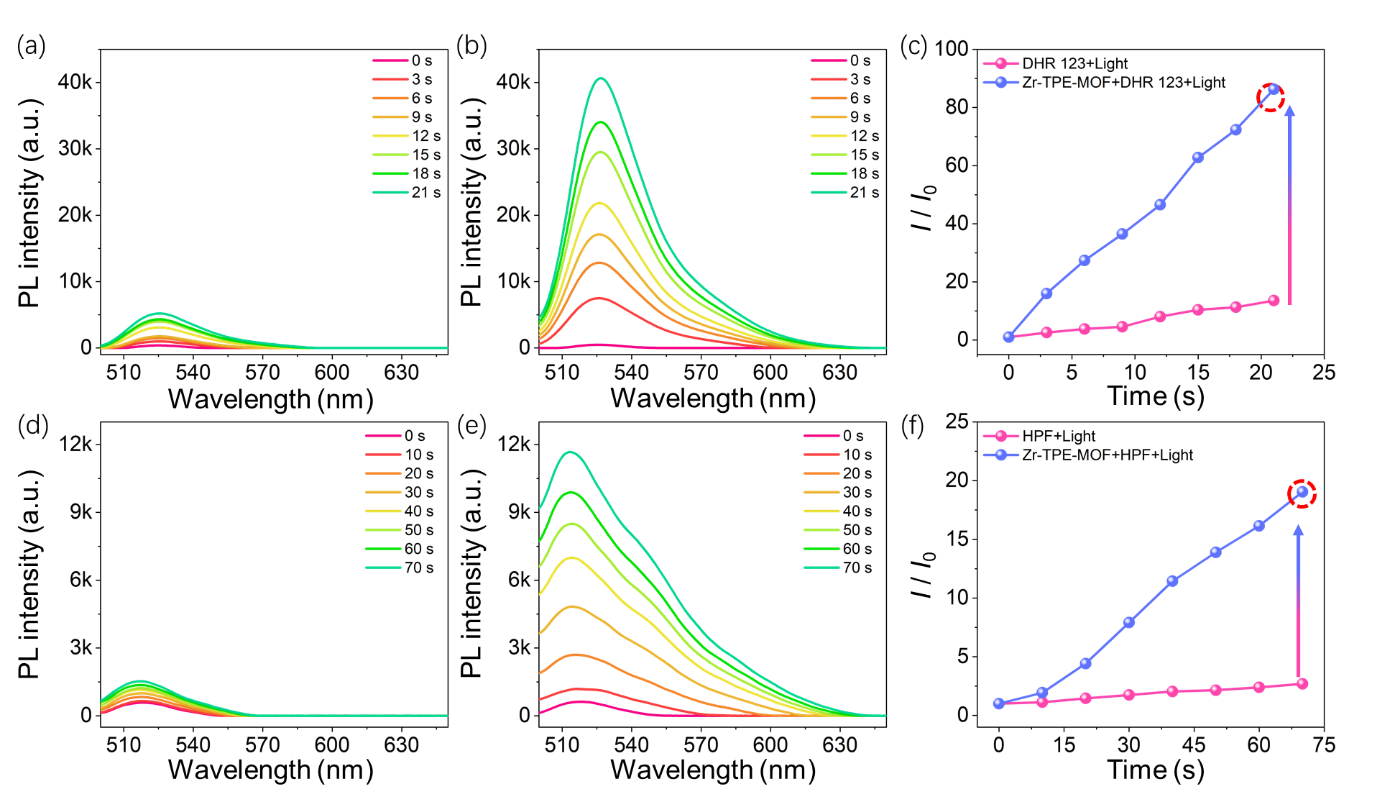


**Figure S13.** (a and d) Fluorescence spectra of aqueous solutions containing only DHR 123 and HPF (20 μM) under different illumination times (60 mW·cm^−2^); (b and e) Fluorescence spectra of aqueous solutions containing DHR 123 and HPF (20 μM) with a concentration of 10 μg·mL^−1^ Zr-TPE-MOF under different illumination times (60 mW·cm^−2^); (c and f) ·O_2_^−^ and ·OH generation capacities of Zr-TPE-MOF using DHR 123 and HPF as indicators, respectively.


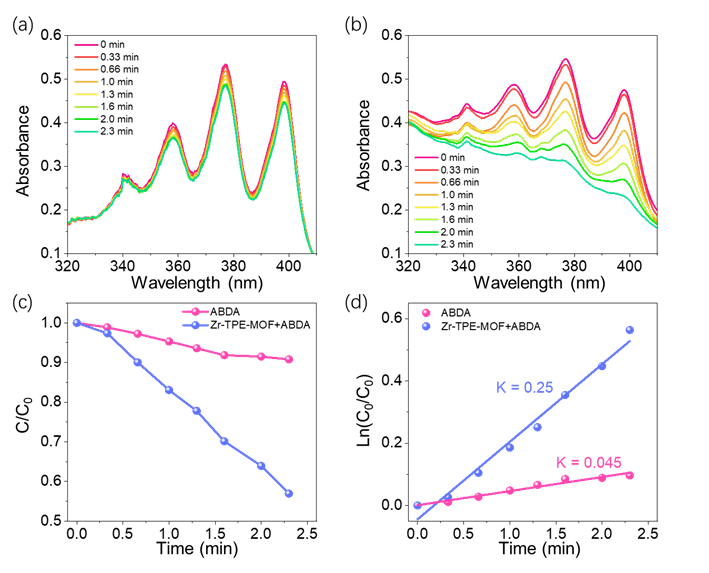


**Figure S14.** (a) Absorption spectra change of aqueous solution containing only ABDA (0.1 mM) after light irradiation (60 mW·cm^−2^) for different time periods; Absorption spectra change of aqueous solution containing ABDA (0.1 mM) containing Zr-TPE-MOF (b) with increasing light irradiation (60 mW·cm^−2^) time; ^1^O_2_ generation capacities and rates of Zr-TPE-MOF using ABDA as the indicator.


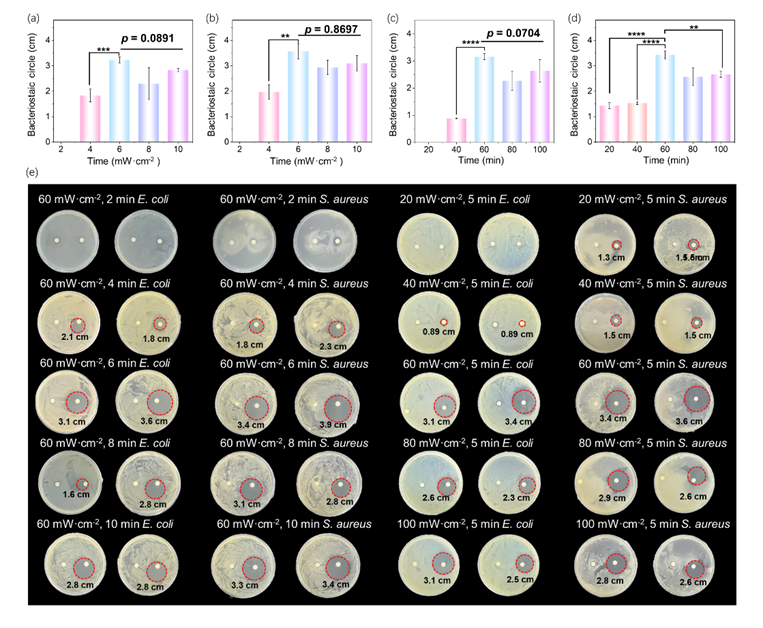


**Figure S15.** (a-d) The size of the inhibition zone of Zr-TPE-MOF against *E. coli* and *S. aureus* under different illumination time and power conditions; (e) Photodynamic bactericidal effect of Zr-TPE-MOF on *E. coli* and *S. aureus* under different illumination time and power conditions (Repeat 3 times, *n* = 3). (Note: Data represent mean ± SD. *n* = 3, (a) ***p < 0.0009 and No significant difference, p = 0.0891; (b) ***p* < 0.0025 and No significant difference, *p* = 0.08697; (c) *****p* < 0.0001 and No significant difference, *p* = 0.0704; (d) *****p* < 0.0001 and ***p* < 0.0070.)


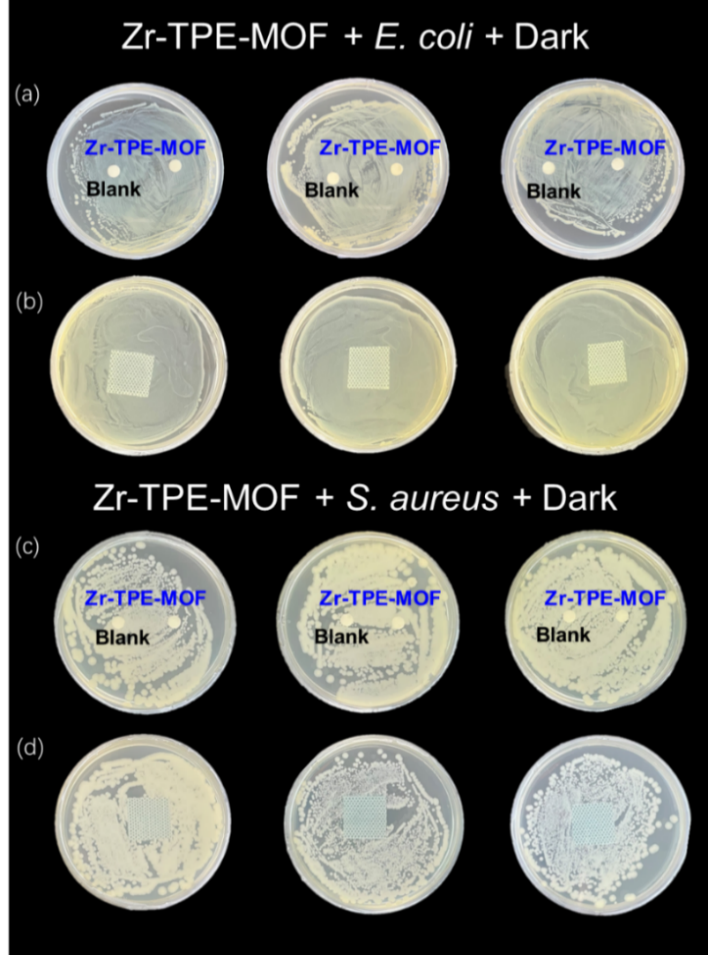


**Figure S16.** Photos of the antibacterial effect of Zr-TPE-MOF (3 mg·mL^−1^) on *E. coli* and *S.* *aureus* under dark (blank) and light conditions (60 mW·cm^−2^, 5 min) (Repeat 3 times, *n* = 3).


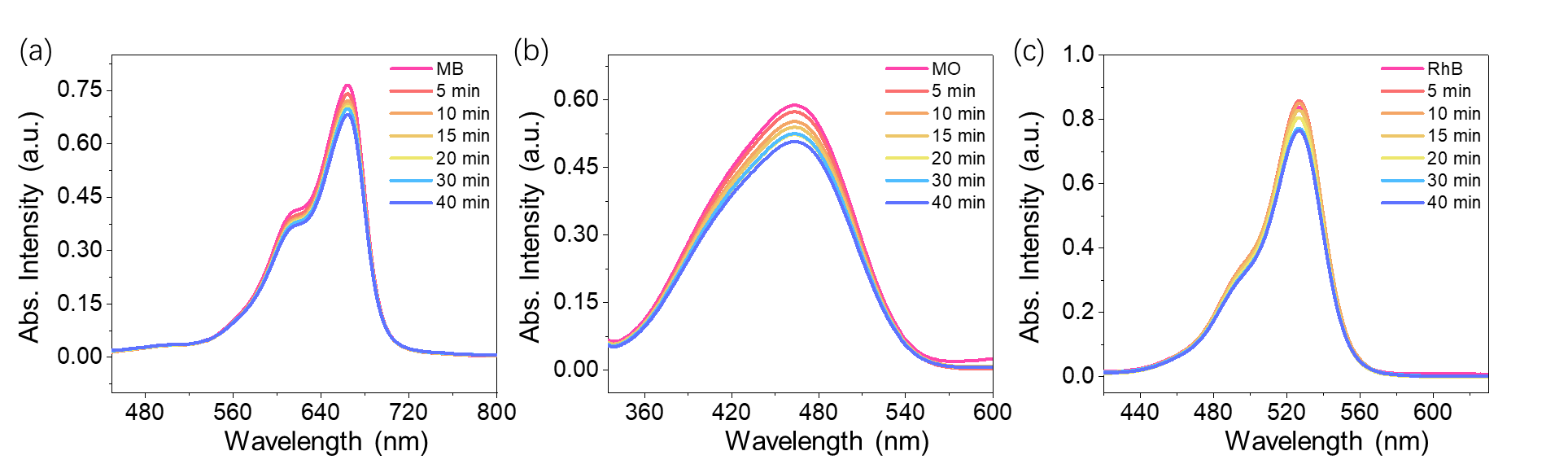


**Figure S17.** UV–Vis absorption spectra of organic dye (MB = MO = RhB = 10 mg·L^−1^) aqueous solutions (pH = 7.0) under different illumination times (60 mW·cm^-2^).


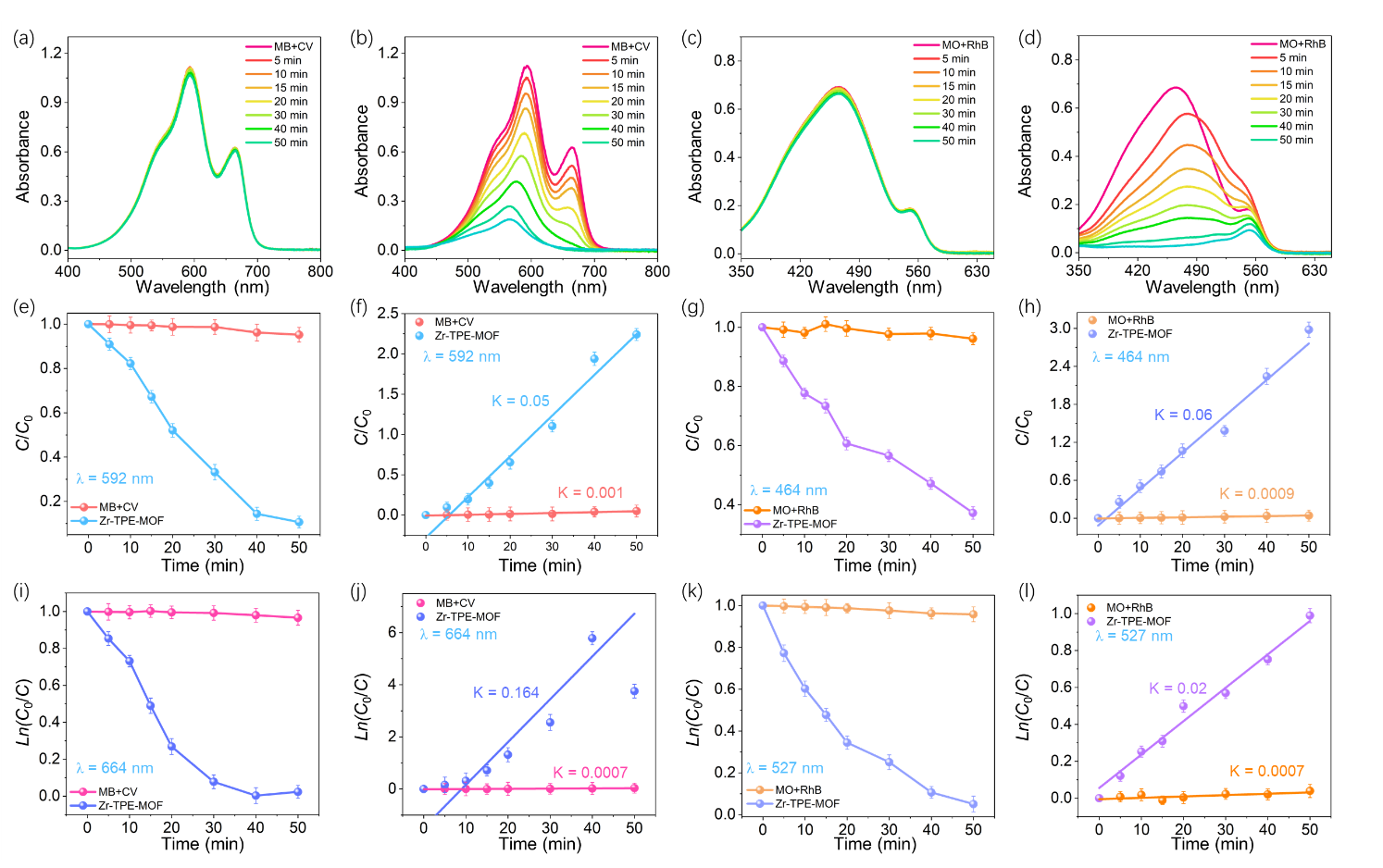


**Figure S18.** (a and b) UV-visible absorption spectra of mixed dye aqueous solutions of CV (10 mg·L^−1^) and MB (10 mg·L^−1^) in the absence and presence of Zr-TPE-MOF (0.3 mg·mL^−1^) as a function of light irradiation time; (c and d) UV-visible absorption spectra of mixed dye aqueous solutions of MO (10 mg·L^−1^) and RhB (10 mg·L^−1^) in the absence and presence of Zr-TPE-MOF (0.3 mg·mL^−1^) as a function of light irradiation time (Repeat 3 times, n = 3); Degradation curves (e-h) and degradation kinetic fitting diagrams (i-l) of the above Zr-TPE-MOF (0.3 mg·mL^−1^) degradation of the mixed dye system (Repeat 3 times, *n* = 3).


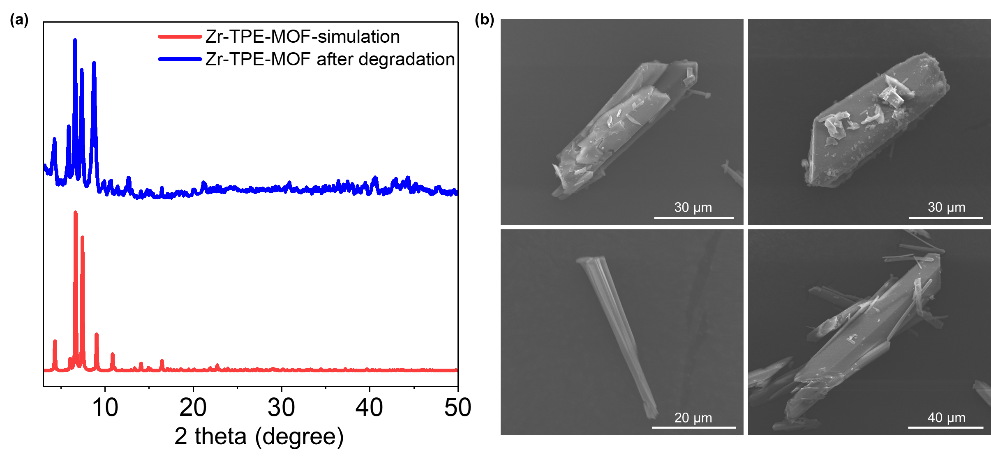


**Figure S19.** (a) PXRD of Zr-TPE-MOF after photocatalytic degradation of organic dye aqueous; (b) SEM of Zr-TPE-MOF after photocatalytic degradation of organic dye aqueous.

**References**

1. Sheldrick, G. M. *Acta Crystallogr., Sect. C: Struct. Chem*. **2015**, *71*, 3–8.
2. Zhu, Z.-H.; Liu, Y.; Song, C.; Hu, Y.; Feng, G.; Tang, B. Z. Porphyrin-Based Two-Dimensional Layered Metal–Organic Framework with Sono-/Photocatalytic Activity for Water Decontamination. *ACS Nano* **2022**, *16*, 1346–1357.
3. Gan, S.; Wu, W.; Feng, G.; Wang, Z.; Liu, B.; Tang, B. Z. Size Optimization of Organic Nanoparticles with Aggregation‐Induced Emission Characteristics for Improved ROS Generation and Photodynamic Cancer Cell Ablation. *Small* **2022**, *18*, 2202242.
4. Zhu, Z. H.; Zhang, L.; Jia, S.; Ni, Z.; Li, Y. L.; Zou, H. H.; Yang, Y.; Hu, Y.; Ding, D.; Tang, B. Z.; Feng, G. Nanoscale Metal-Organic Framework Leveraging Water, Oxygen, and Hydron Peroxide to Generate Reactive Oxygen Species for Cancer Therapy. *Adv. Funct. Mater.* **2025**, 2419548.
5. Zhu, Z. H.; Zhang, D.; Chen, J.; Zou, H. H.; Ni, Z.; Yu, Y.; Hu, Y.; Liu, R.; Feng, G.; Tang, B. Z. A Cascade Strategy Boosting Hydroxyl Radical Generation with Aggregation-Induced Emission Photosensitizers-Albumin Complex for Photodynamic Therapy. *ACS Nano* **2023**, *17*, 16993–17003.
